# Genotyping by Sequencing Advancements in Barley

**DOI:** 10.3389/fpls.2022.931423

**Published:** 2022-08-08

**Authors:** Nirmal Raj Rajendran, Naeela Qureshi, Mohammad Pourkheirandish

**Affiliations:** ^1^Faculty of Veterinary and Agricultural Sciences, University of Melbourne, Parkville, VIC, Australia; ^2^International Maize and Wheat Improvement Center (CIMMYT), El Batan, Texcoco, Estado de Mexico, Mexico

**Keywords:** barley, genotyping by sequencing, restriction enzymes, target enrichment, SNPs

## Abstract

Barley is considered an ideal crop to study cereal genetics due to its close relationship with wheat and diploid ancestral genome. It plays a crucial role in reducing risks to global food security posed by climate change. Genetic variations in the traits of interest in crops are vital for their improvement. DNA markers have been widely used to estimate these variations in populations. With the advancements in next-generation sequencing, breeders could access different types of genetic variations within different lines, with single-nucleotide polymorphisms (SNPs) being the most common type. However, genotyping barley with whole genome sequencing (WGS) is challenged by the higher cost and computational demand caused by the large genome size (5.5GB) and a high proportion of repetitive sequences (80%). Genotyping-by-sequencing (GBS) protocols based on restriction enzymes and target enrichment allow a cost-effective SNP discovery by reducing the genome complexity. In general, GBS has opened up new horizons for plant breeding and genetics. Though considered a reliable alternative to WGS, GBS also presents various computational difficulties, but GBS-specific pipelines are designed to overcome these challenges. Moreover, a robust design for GBS can facilitate the imputation to the WGS level of crops with high linkage disequilibrium. The complete exploitation of GBS advancements will pave the way to a better understanding of crop genetics and offer opportunities for the successful improvement of barley and its close relatives.

## Introduction

Barley, *Hordeum vulgare*, is one of the earliest domesticated crop species that has played a critical role in the development of human civilization (Pankin and Von Korff, 2017). It is the fourth most important cultivated cereal crop globally (Pham et al., 2019), produced mainly for livestock feeding and the brewing industry (Ullrich, 2010). Barley is an ideal model crop for the tribe *Triticeae* due to its diploid nature, the low number of chromosomes (*n* = 7), close relationship with wheat, and a wide diversity with no crossing barriers between cultivated forms and their wild progenitors (Pourkheirandish and Komatsuda, 2007; Romero et al., 2018; Harwood, 2019). There are approximately 400,000 *Hordeum* accessions registered in various genebanks around the world that serve as a valuable resource for crop breeding to solve the important global challenges in agriculture (Knüpffer, 2009; FAO, 2010; Galluzzi et al., 2020). A robust barley reference genome with high-quality annotation and a pan-genome assembly with novel genetic variations is now publicly available, enabling the analysis of high-throughput sequencing data (Mascher et al., 2017; Jayakodi et al., 2020).

In the wake of unprecedented climate change and decline in the area of arable land, crop improvement has become an uphill task for plant breeders (Wang et al., 2019). Crop management and plant breeding strategies offer large potential to tackle present and future challenges of crop production (Wang et al., 2015). Breeding for combining desirable traits is the most sustainable, economical and efficient way of crop improvement. Integrating precise high-throughput phenotyping approaches with genome sequencing allows the identification of critical genomic regions controlling important agronomic traits. The selection of cultivars for traits of interest based on DNA markers is known as marker-assisted selection (MAS). With the development and easy access to a wide range of DNA markers and genetic maps, it is now possible to carry out MAS for traits governed by major as well as minor genes called quantitative trait loci (QTL) (Babu et al., 2004). Molecular markers are not affected by environmental factors, and selection can be performed at the early stages of plant development (Hasan et al., 2021). For the successful application of MAS, the precise location of QTL, the availability of closely linked markers and the effect of QTL within the genome must be studied in advance (Wang et al., 2016). Once a close linkage disequilibrium between a trait and molecular markers is established, the markers can be used to select desirable traits during breeding cycles (Vafadar Shamasbi et al., 2017; Bhavani et al., 2021). MAS becomes highly unreliable when dealing with complex quantitative traits regulated by many minor QTLs. The effects of these minor QTLs determined by linkage mapping and genome-wide association mapping is limited due to its reliance on statistical power, hence require a very large population to identify the desirable QTL combinations (Bhat et al., 2016).

Molecular markers can be categorized into three classes based on their mechanism of detection: hybridization, polymerase chain reaction (PCR) and sequencing. Hybridization-based molecular markers require a segment of DNA known as a probe to identify an individual. In plants, restriction fragment length polymorphism (RFLP) was the first-generation of hybridization-based molecular markers. The application of RFLP was very limited due to its low-throughput nature, difficulties in performing hybridization and the low polymorphism ratio due to the use of restriction enzymes at random. The hybridization-based markers were gradually replaced by the more efficient and inexpensive PCR-based tags, in which a specific segment of DNA is multiplied millions of times (Singh and Singh, 2015). There are many types of PCR-based markers, including randomly amplified polymorphic DNA (RAPD), sequence characterized amplified region (SCAR), cleaved amplified polymorphic sequence (CAPS), inter-simple sequence repeats (ISSRs), amplified fragment length polymorphism (AFLP) and simple sequence repeats (SSRs). Among these, SSRs have proved to be one of the most efficient markers as they are present in abundance to detect a high level of polymorphism (Deschamps et al., 2012). Most PCR-based molecular markers were traditionally designed or identified based on a minimal set of sequence data. Recently, sequencing-based markers have gained traction since variations at single-nucleotide resolution can be directly selected and effectively utilized as genetic markers, owing to their abundance in all species (Garrido-Cardenas et al., 2018).

Scientists employed Sanger Sequencing method in many projects advancing the era of sequencing-based markers, including expressed sequence tags (ESTs) which proved to be a steppingstone to identify single-nucleotide polymorphisms (SNPs) in the expressed region of the genome (Wang et al., 1998). However, due to high cost and low throughput, Sanger sequencing is replaced by high throughput and more cost-efficient approaches including hybridization-based microarrays and next-generation sequencing (NGS) technologies. The hybridization-based microarrays are performed by designing thousands of probes based on the available sequence data on a small chip to detect polymorphisms. Microarray-based high-throughput SNP genotyping platforms have been developed over the years and many commercial platforms are now available such as, Illumina Infinium II, Axiom BioBank and Ion Torrent (Gupta et al., 2008; Phan and Sim, 2017). In barley, an array-based platform with 1,572 SNP markers was first introduced in 2006 with the development of Illumina GoldenGate assays (Close et al., 2009). The 9 k Illumina Infinium iSelect BeadChip succeeded the GoldenGate assays in 2009 (Comadran et al., 2012). The latest addition to this line of high-throughput genotyping platform is the 50 k Illumina Infinium iSelect SNP array (Bayer et al., 2017). These SNP markers are now being widely used in genome-wide studies (Nielsen et al., 2011).

Researchers often use NGS techniques on a large scale to assess genetic variations in a wide range of crops (Poland and Rife, 2012). Genome sequencing protocols have been developed in combination with bioinformatics procedures, including SNP marker discovery and genotyping. The most popular technologies are multiplexed shotgun sequencing (MSG), restriction enzyme based genotyping by sequencing (GBS) and whole genome sequencing (WGS) to access sequence variation in the studied species (Yang et al., 2017). In general, GBS has been referred to all those approaches aiming to construct a reduced genome representation libraries for sequencing. There has been a growing preference for using reduced genome sequencing protocols rather than whole genome sequencing, especially for crops with large genome size and high levels of linkage disequilibrium due to cost effectiveness, with an efficient sequence analysis performed in crops even without a robust reference sequence, often referred to as *de novo* sequencing (Lu et al., 2015; Darrier et al., 2019). However, the application of GBS over other genotyping approaches such as, array-based genotyping and WGS depends upon the research objectives, resource availability, timeframe of project and the skillset of the personnel’s involved. The cost effectiveness can be mainly attributed to the simplified genome, the inexpensive barcoding system used for multiplexing in every GBS strategy (He et al., 2014), and the increased sequencing capacity per run in platforms. The SNP data generated by the GBS approach can now be easily analyzed using plenty of well-established bioinformatics pipelines to filter SNP markers with precision (Raman et al., 2014). High-density genetic maps constructed with such SNP markers are better than those built by traditional microsatellite markers (Mayer et al., 2012). SNPs of interest generated by the GBS platforms can be verified by converting them into PCR-based tags, followed by marker validation, which facilitates the genotyping for large populations without the need for repeating the whole GBS assay (Yang et al., 2017). Given the size of the barley genome (5.5GB), it is essential to optimize the sequencing protocols to overcome genome complexity. The primary focus of this review article is to put forward ideas about the GBS based protocols that are currently used and have the potential to be used for barley improvement in the future.

## Barley Genomic Resources

### Germplasm Collections

Barley germplasm collection, including cultivars, landraces, advanced breeding materials, genetic stocks, hybrids, induced mutant lines and wild relatives, established in various plant Genebanks, provides precious genetic resources for breeding and research programs worldwide (Table 1). The total number of accessions conserved in Genebanks can be found by browsing through online databases, such as FAO WIEWS^1^ and Genesys.^2^ Barley accessions from collected materials, ranks second among the cereal crops, after wheat (Kant et al., 2016). As of March 2022, in the world’s largest Svalbard Global seed vault, managed by Nordic Genetic Resource Center (NordGen), Sweden, there are 106,887 accessions of the genus *Hordeum*.^3^ The most extensive collection of barley germplasm accessions is in Plant Gene Resources of Canada, followed by the National Small Grains Germplasm Research Facility in the United States. Another main center of worldwide barley collection is in the International Centre for Agricultural Research in Dry Areas (ICARDA), Lebanon, which maintains wild and cultivated barley accessions originating from different regions across the globe (Kant et al., 2016). Barley genetic resources representing the South and East Asian regions are available in Japan. The University of Okayama in Japan maintains a subset of international barley core collection with 380 lines (Sato, 2020). Additionally, there are mapping populations registered to be used by researchers. For example, three different mapping populations *viz*., Steptoe × Morex doubled haploids (DHs), Harrington × Morex DHs and Oregon Wolfe Barley DHs (Kleinhofs et al., 1993; Marquez-Cedillo et al., 2000; Szűcs et al., 2009) developed under the North American Barley Genome Mapping Project (NABGMP) have been deposited in the Okayama University. These mapping populations are crucial for the development of high-density consensus genetic maps and identifying QTLs for prioritized traits in barley (Sato, 2020). Apart from these conventional approaches, researchers have produced a cross between cultivated barley and *Hordeum bulbosum*, a wild self-incompatible relative from the secondary gene pool of barley to develop a set of *H. bulbosum* introgression lines (Pickering, 1984; Pickering et al., 1995). These introgression lines harbor a wide range of resistant traits that have been used to identify resistant genes for various diseases like leaf rust (Yu et al., 2018), mild mosaic virus (Ruge-Wehling et al., 2006), yellow dwarf virus (Scholz et al., 2009) and powdery mildew (Hoseinzadeh et al., 2020).

**Table 1:** Barley accessions present in major gene banks (FAO WIEWS, 2022; Genesys, 2022).

S/No.	Major countries	Total no. of accessions	Major gene bank	FAO id	URL
1	Canada	41,475	Plant Gene Resources of Canada, Saskatoon Research and Development Centre	CAN004	http://pgrc.agr.gc.ca/
2	United States	37,355	National Small Grains Germplasm Research Facility, USDA-ARS	USA029	http://www.ars.usda.gov/main/docs.htm?docid=2884
3	Lebanon	32,451	International Centre for Agricultural Research in Dry Areas	LBN002	https://www.icarda.org/
4	Germany	21,956	Leibniz Institute of Plant Genetics and Crop Plant Research	DEU146	http://www.ipk-gatersleben.de
5	Brazil	20,868	Embrapa Recursos Genéticos e Biotecnología	BRA003	https://www.embrapa.br/recursos-geneticos-e-biotecnologia
6	Australia	19,064	Australian Grains Genebank, Agriculture Victoria	AUS165	–
7	Russian Federation	17,788	NI Vavilov Research Institute of Plant Industry	RUS001	http://www.vir.nw.ru
8	Sweden	16,638	Nordic Genetic Resource Center	SWE054	http://www.nordgen.org
9	Japan	15,777	NARO Genebank	JPN183	http://www.gene.affrc.go.jp/about_en.php
10	Mexico	15,330	Centro Internacional de Mejoramiento de Maíz y Trigo (CIMMYT)	MEX002	http://www.cimmyt.org/
11	Ethiopia	16,612	Ethiopian Bio-diversity Institute	ETH085	http://www.ebi.gov.et
12	United Kingdom	10,925	Germplasm Resources Unit, John Innes Centre, Norwich Research Park	GBR247	http://www.jic.ac.uk/germplasm/
13	India	8,601	National Bureau of Plant Genetic Resources	IND001	http://www.nbpgr.ernet.in

The research on barley mutations started in1928 (Ohnoutkova, 2019). The mutants produced are preserved at the Nordic Genetic Resource Centre and the United States Department of Agriculture (USDA) National Small Grain Collection (Lundqvist and Franckowiak, 2003). The mutants are grouped into different phenotypic categories, labeled with a UL prefix (Lundqvist and SvalöfWeibull, 2005), and can be accessed through the International Database for Barley Genes and Barley Genetic Stocks.^4^ This database currently lists 754 barley genetic stocks with detailed description. Several researchers have used these mutants in genetic studies and for gene identification (Komatsuda et al., 2007; Li et al., 2013, 2017).

### Genomic Resources

Since the beginning of 21st century, EST sequencing projects have gained momentum in crops, including barley, allowing functional characterization of genomic sequences (Sato, 2020). High-resolution EST maps with 1,032 EST based loci and 2890 EST based loci were constructed by Stein et al. (2007) and Sato et al. (2009), respectively. The six-row American malting cultivar known as Morex (reference genotype) has been utilized to construct bacterial artificial chromosome (BAC) libraries, that have been helpful in creating the physical map with few gaps (Yu et al., 2000; Schulte et al., 2011). The International Barley Genome Sequencing Consortium (IBSC) was established in 2006 to construct a high-quality genome sequence by consolidating all the available genomic resources (Schulte et al., 2009), and later the Leibniz Institute of Plant Genetics and Crop Plant Research (IPK)^5^ launched a search tool to run BLAST against the barley genome sequence compiled by IBSC. Similarly, the EnsemblPlants portal also allows researchers to browse and blast search on the target sequence. The first generation of chromosome-scale barley pan-genome assembly have been obtained from a core set of 20 diverse barley genotypes (Jayakodi et al., 2020) and this has given the researchers some insights on the effect of structural variants at single base resolution in barley evolution (Kamal et al., 2022). A complete transcriptome profile for the Morex reference genome, which is essential for the annotation of available genome assemblies, is also available, in addition to its genomic sequences. A reference transcript dataset (BaRTv1.0) consisting of 60,444 genes covering various organs and tissues was also developed (Rapazote-Flores et al., 2019) and a set of transcript sequences from RNA-Seq is catalogued in an open-access genome database barleyGenes.^6^ Recently, a barley expression database known as EORNA^7^ provided a single-window platform for researchers to visualize variations in gene expression profiles across different barley genotypes (Milne et al., 2021).

## Genotyping by Sequencing

GBS has become a widely adopted high-throughput sequencing approach to mine the potential high value SNPs and small insertion/deletion variations in species with complex genomes, such as wheat and barley when compared to whole genome sequencing and the array-based SNP genotyping (Bajgain et al., 2016). Though SNPs are efficient, the application of whole genome sequencing for their detection is costly and cumbersome, especially in crops with a complex genome structure. A complex genome has highly repetitive regions, duplicative DNA sequence and polyploidy, posing problems during sequence assembly and SNP detection (Mardis, 2008; Mammadov et al., 2012). Among cereals, wheat (16 GB) and barley (5.5 GB) have large complex genomes with over 80% of repetitive elements (Wicker et al., 2009). Hence, sequencing has not always been a straightforward approach for these crops, and thus a higher sequencing depth is required to achieve SNP calling (Dou et al., 2012; Jighly, 2022). To overcome these challenges in SNP identification, the genome size reduction was introduced as a low-depth sequencing strategy (Huang et al., 2009; Scheben et al., 2017). Also, exponential growth in NGS have simplified GBS protocols to mine thousands of SNPs covering a substantial portion of the barley genome (Alqudah et al., 2020).

The reduced genome representation strategies being successfully adopted in barley are generally divided into two classes. The first class involves techniques that rely on restriction enzyme (RE) cut sites to generate fragments for the construction of sequencing libraries. Genome reduction based on RE cut sites ensures consistency in the sequenced portion of genome in individuals as RE sites are generally conserved across species (Chung et al., 2017). Moreover, the use of methylation-sensitive REs in GBS provides a high SNP coverage in gene-rich regions of the genome rather than in repetitive inter-genic regions (Fellers, 2008). For example, methylation-sensitive REs cannot cleave methylated cytosine residues, and gene-rich regions exhibit very low levels of cytosine methylation in plants (Zhang et al., 2010). In gene-rich regions, SNP markers are usually preferred due to the nature of unique sequences in genic regions and the probability of finding a desirable QTL in the vicinity (Pootakham et al., 2016). The second class of GBS for genome reduction is sequencing targeted regions in the genome, that could involve various approaches, such as multiplex PCR assay for genes or genomic subsets (Tewhey et al., 2009), hybridization-based sequence capture (Gnirke et al., 2009), molecular inversion probes (MIPs) (Hardenbol et al., 2005) RNA sequencing (RNA-Seq) for transcriptome profiling, exome capture to isolate the coding sequence (exon) variants, deoxyribonuclease I (DNase I)-hypersensitive site sequencing (DNase-Seq) to determine chromatin accessibility and chromatin immunoprecipitation sequencing (ChIP-Seq) to analyze protein-DNA interactions. Several target enrichment methods for NGS have also been developed, which provide the highest degree of control for the identification of targeted genomic regions for SNP discovery in a cost-effective and time-efficient manner.

**Figure 1:**
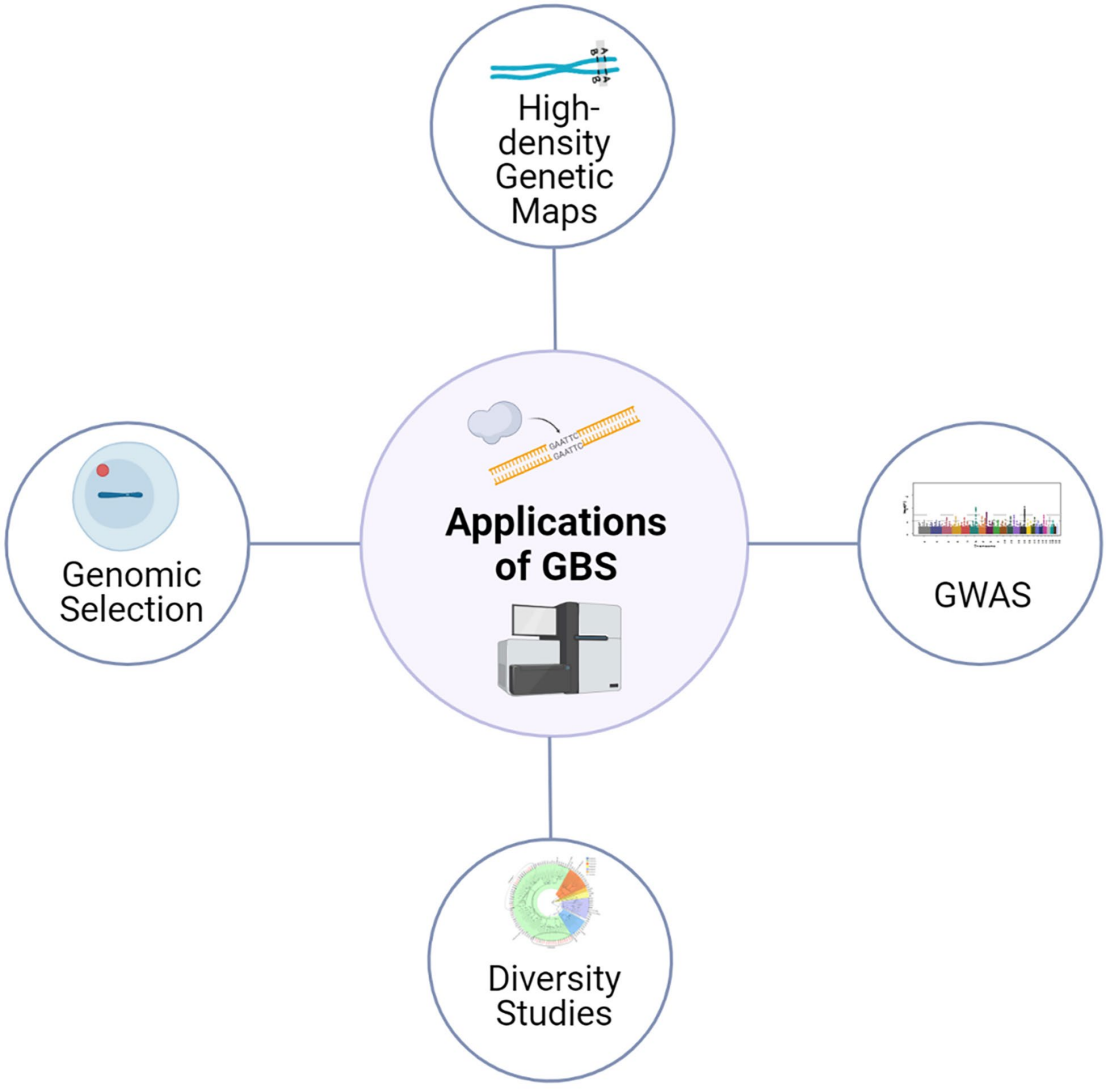
Application of GBS approaches for various genetics and plant breeding studies. The high-quality SNPs derived from GBS are being used for diversity analysis, genetic map construction, genome-wide association studies and genomic selection.

The reduced genome GBS approaches based on REs can also be applied in species without a reference genome (*de novo*) and also without any prior SNP information (Rasheed et al., 2017). In *de novo* approaches, first, GBS sequence data is processed based on identical raw reads; a bioinformatic pipeline would then identify other highly similar reads that are probably from the same location. The rest of the sequence reads without high similarity are usually discarded from the analysis. However, in the presence of a robust reference sequence assembly, GBS data is directly aligned against the reference sequence, and most sequence reads could be used to discover polymorphisms (Kim et al., 2019). Currently, reference quality genomes are available for many crops, including barley, alongside extensive data from well characterized collections of SNPs (Chung et al., 2017; Alqudah et al., 2020); thus, making this approach widely applicable for crop genetics and plant breeding research, with high confidence (Figure 1).

### Genotyping by Sequencing Based on REs

**Figure 2:**
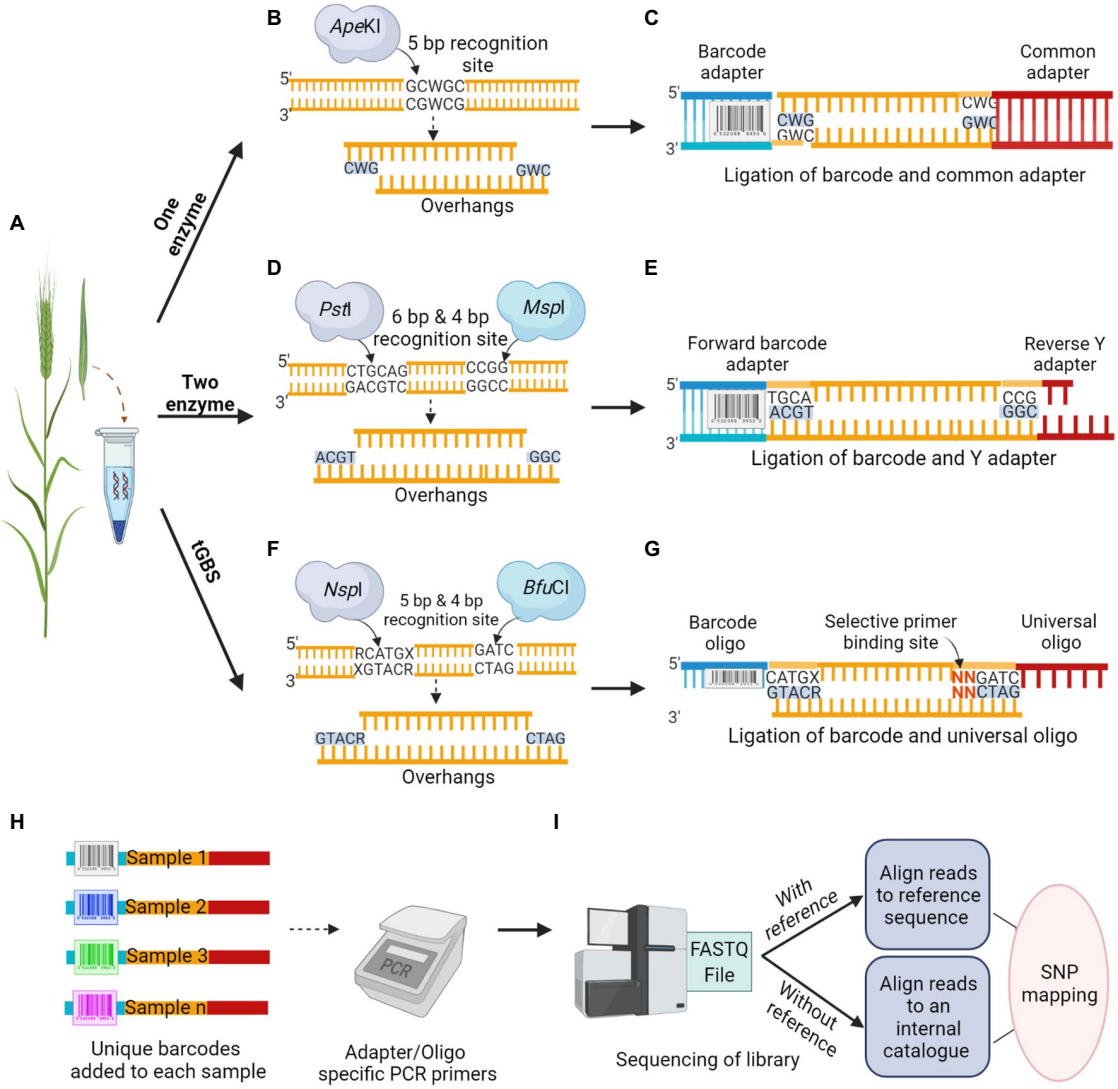
Schematic overview of Restriction Enzyme based GBS methodology. **(A)** Tissue sample collection is followed by DNA isolation from the crop. **(B,D,F)** DNA digestion by; One enzyme, *Ape*KI, that makes a cut to produce overhangs **(B)**, classical Two enzyme, a rare cutter *Pst*I and a common cutter *Msp*I produces overhangs **(D)**, and new version of Two enzyme called tGBS, using a different set of restriction enzymes, *Nsp*I and *Bfu*CI capable of better digestion produces overhangs in opposite directions **(F)**. **(C,E,G)** Adapter ligation to the digested sample; One enzyme, ligation of barcode and common adapter **(C)**, classical Two enzyme, ligation of forward barcode and reverse Y adapter **(E)**, tGBS, ligation of barcode and universal oligo. Nucleotide sequence in red possesses the matching bases in selective primer for target specificity **(G)**. **(H)** Multiplexing; a unique barcode used for each accession allows the pooling of DNA samples before the amplification step. **(I)** The mixed libraries, uniquely barcoded, and amplified samples will be run in a next-generation sequencer. The sequence reads will be analyzed in various bioinformatic pipelines based on the availability of reference sequence.

The first step in GBS is to construct a robust library, the most critical step before any sequencing process, which determines the overall quality and coverage of reads and affects the achieved sequencing depth. The construction of the GBS library involves two major steps, including the RE based genome reduction, followed by multiplexing samples using barcode adapters or oligos. This approach is simple, fast, unique and reproducible that can even reach the genomic regions of interest, which were previously inaccessible to sequence capture approaches (Elshire et al., 2011). The importance of restriction site associated genomic DNA sequencing for SNP discovery and genotyping was first demonstrated by Baird et al. (2008). Multiplex sequencing by adding inexpensive DNA barcodes in species with small genomes like rice (0.43 GB), with a genome size 10 times smaller than that of barley, was suggested by Craig et al. (2008). Since then, it has become a standard practice in most Illumina supported NGS applications. Simple multiplexing is easily accessible for crops with small genome sizes, whereas in crops with large genome sizes, using a combination of two techniques, involving RE for genome reduction and subsequent multiplexing is required to achieve high-throughput and reliable sequencing (Elshire et al., 2011). The RE targets low-copy genomic regions, thereby minimizing repetitive sequences from sequence reads. The GBS based on REs has several versions according to the number of restriction enzymes used, along with the type of adapters ligated to DNA fragments (Figure 2).

#### One Enzyme GBS

One enzyme GBS approach is one of the first-generation techniques for genome reduction developed by Elshire et al. (2011), with a straightforward protocol widely used in breeding and trait mapping (Goddard et al., 2019). This method was first applied in both maize and barley, with the selection of REs as a key factor in determining coverage. A methylation-sensitive 5 bp cutter, *Ape*KI was suggested as an ideal RE, which significantly reduced the repetitive sequence in barley and maize genomes (Figure 2B). The overhangs generated by the RE would anchor the adapter ligation to the intended DNA sequence. A double-stranded universal and barcoded adapter were ligated to digested DNA samples (Figure 2C). A different barcoded adapter is considered for each sample, allowing multiplexing of individuals per sequencing lane during a single run to significantly reduce genotyping cost. The adapters were designed to avoid restriction enzyme recognition sites and no regeneration of the recognition site should occur after ligation of genomic DNA fragments. Unlike earlier RE based library construction, where RE generated DNA fragments were too large for sequencing; thus, requiring further fragmentation and size selection, one enzyme GBS generates fragments suitable for NGS. This allows digestion and adapter ligation to proceed in a single well and direct sequencing (Elshire et al., 2011; Wickland et al., 2017). Digested fragments from the libraries were amplified in a PCR reaction with primer sets that are complementary to the ligated adapters before sequencing. The sequencing of the GBS library can be performed in a variety of NGS platforms, such as Illumina Genome Analyzer II (48/96 plex). Using the one enzyme approach, 24,186 sequence tags were mapped onto the Oregon Wolfe Barley genetic map (Elshire et al., 2011). With the consensus read sequence associated with restriction sites as reference from this GBS approach, the mapping can be performed to uncover genetic variations even without a reference genome. In the one enzyme GBS approach, a non-uniform library was constructed due to biases associated with a single RE targeting a single cut site, and there was a scope for further reduction in genome complexity.

#### Two Enzyme GBS

To construct a library with a greater degree of genome reduction and uniformity, the two enzyme GBS approach was introduced as a successor, which was successfully demonstrated in both barley and wheat (Poland et al., 2012). In this approach, a rare cutter with six base recognition sites (*Pst*I) in combination with a common cutter with four base recognition sites (*Msp*I) were used. Two adapters were designed for *Pst*I and *Msp*I restriction overhangs (Figure 2D); the forward adapter with a barcode for the rare cut site and the universal reverse Y adapter for the common cut site (Figure 2E). In this approach, each fragment in the library has two different adapters, forward and reverse, in the same orientation as the cut sites. The digestion by two enzymes would result in DNA fragments with both rare and common cut sites whereas, fragments with only common cut sites and rare cut sites would be in extremely low frequency. To avoid the amplification of fragments with two common cut sites and adapter dimers, the universal Y shaped adapter was designed as a reverse adapter. The Y adapters introduced by Illumina ensures that each DNA fragment is only ligated to different adapter pairs (Bentley et al., 2008). During the first round of PCR amplification, only the forward primer can anneal to the forward barcode adapter, whereas the reverse primer cannot anneal during this time as the tail sequence of the reverse Y adapter is not a complement of the reverse primer. The complementary sequence of the reverse primer was only generated at the end of the first round of PCR, ensuring the DNA amplification of only *Pst*I–*Msp*I fragments. The fragments with the same adapters on each end of the fragment would inhibit the formation of library clusters on the sequencing flow cell as they cannot undergo bridge amplification, leading to the loss of sequencing efficiency for segments. The two enzyme approach could ensure the uniformity of sequences from forward to reverse direction but not vice versa, which led to an increase in efficiency while representing the subset of a whole genome. The analysis of 82 Oregon Wolfe Barley DH populations using the Illumina Genome Analyzer II (48 plex) identified approximately 34,000 SNPs and 24,159 tags with less than 20% missing data (Poland et al., 2012).

Both one enzyme and two enzyme GBS approaches have been frequently used for linkage mapping analysis to identify QTLs for agronomic traits (Scheben et al., 2017) due to their simplicity, efficiency and cost effectiveness. However, these approaches produced highly skewed coverage of genomic positions (Beissinger et al., 2013). Moreover, they produce low read depth per locus when common cutting REs are used to produce small fragments (Pootakham et al., 2016) and fragmentation is confined to RE sites, with some genomic regions not being sequenced, it leads to more missing data (missing a genotype value at loci). Low read depths are likely responsible for the ineffective representation of heterozygous loci (Torkamaneh et al., 2016). To correct the errors associated with heterozygous loci, SNP calls from these approaches were considered as missing data or as dominant markers during GBS data analysis. However, these errors do not cause major problems in barley or wheat since they are self-pollinated. The GBS missing data had a significant impact during genetic mapping, as specific SNPs were placed in more than one position on a map. Such variable proportions of shared loci would result in low call rates per sample (Dacosta and Sorenson, 2014). One way to deal with missing data is to avoid SNP loci with missing data altogether; however, this would lead to reduction in the number of available SNPs. Another viable option is to use data imputation, in which a predicted allelic dosage would be substituted with the missing data (Torkamaneh and Belzile, 2015). It is interesting to note that though both one and two enzyme GBS approaches appear to have certain drawbacks associated with low read depth, such as missing data and high error rates at heterozygous loci. However, their robustness would increase with the availability of more efficient imputation algorithms to fill in missing data where the algorithms can function well in the presence or absence of reference genomes (Manching et al., 2017; Munyengwa et al., 2021).

#### Tunable GBS

The shortcomings of both one and two enzyme approaches were addressed by the most recent GBS technology, known as tunable GBS (tGBS^®^). This approach allows a researcher to manipulate the number of targeted genomic sites by merely modifying a single primer in the protocol. Such flexibility in tGBS libraries resulted in higher read depths for each target site compared to the previously mentioned GBS protocols (Ott et al., 2017). Two new sets of REs capable of generating overhangs in opposite directions on the same target strand were employed: a 4 bp cutter, *Bfu*CI, producing a 5′ overhang, and a 5 bp cutter, *Nsp*I, generating a 3′ overhang. These REs achieved a better representation of predicted target sites, and most of the restriction sites were digested in maize (Ott et al., 2017). Two complementary oligos (single-stranded DNA) would be ligated; the oligo complementary to the 3′ overhang possesses a sample-specific barcode sequence, whereas the oligo complementary to the 5′ overhang is universal (Figures 2F,G). After ligation, the samples were subjected to two PCR steps namely, selective PCR and final enrichment PCR. Two primers (selective and non-selective) were specifically designed to ensure their specific annealing to genomic fragments produced during RE digestion. The selective primer was designed to be the reverse complement of the ligated universal oligo, and it can extend up to three nucleotides in the insert sequence at the 3′ end, referred to as selective bases. These selective bases match the digested fragment sequence and enable the primer to precisely select target sites. The non-selective primer was designed to match the region preceding the barcode present in the oligo at the 5′ end. This strategy of ligating unique oligos to overhangs ensure that only the adapter ligated double digested fragments enter the sequencing phase. The replacement of double-stranded adapters with single-stranded oligos in the tGBS^®^ approach enhanced the reliability of library preparation process, as the accurate quantification of oligos is much easier in this method than the quantification of adapters in previous methods. During the final PCR, the sequencing platform-specific primers gets annealed to produce the sequencing library (Ott et al., 2017). To assess the efficiency of tGBS^®^, a study was conducted using both the maize inbred and segregating populations, and sequencing of the constructed library was performed on the Ion Torrent platform. The results revealed that the tGBS^®^ approach was highly efficient in many aspects, especially for the accuracy of SNP calling (>97–98%) at both homozygous and heterozygous sites, compared to the previous GBS approaches. The same DNA library from tGBS® was sequenced in Illumina platform for comparative analysis by using Illumina-specific oligos, which resulted in a similar level of accuracy as reported with the use of the Ion Torrent platform (Ott et al., 2017).

#### Nano-GBS

The advancement of sequencing technologies reduced the cost of high-throughput genotyping (Wetterstrand, 2021); however, the construction of GBS libraries is still the costly phase. A miniaturized protocol for GBS library construction, known as nano-GBS was developed with a reduced cost (Torkamaneh et al., 2020). The new improvisation exploited the non-contact liquid transfer technique (acoustic droplet ejection), which facilitated liquid transfer on a nano-liter scale. The study involved nine libraries constructed with three different combinations of REs (*Ape*KI, *Pst*I*/Msp*I, and *Sbf*I/*Msp*I) and three different multiplexing levels (96 plex, 384 plex, and 768 plex). The library preparation protocol involving one enzyme (*Ape*KI) and two enzyme (*Pst*I*/Msp*I and *Sbf*I/*Msp*I) was based on the works by Elshire et al. (2011) and Abed et al. (2019), respectively. The cost of sequencing was reduced by more than half for genotyping of 96 soybean lines in Ion torrent platform using nano-GBS. Out of three RE combinations, *Pst*I*/Msp*I produced sufficient number of SNPs with a low percentage of missing data. However, the nano-transfer, automation, and the use of multiple combinations of REs are yet to be standardized in many species. The initial installation cost for the nano-transfer equipment is also very high, and the wider adoption of this method could be limited.

### Genotyping by Sequencing Based on Sequence Enrichment

**Figure 3:**
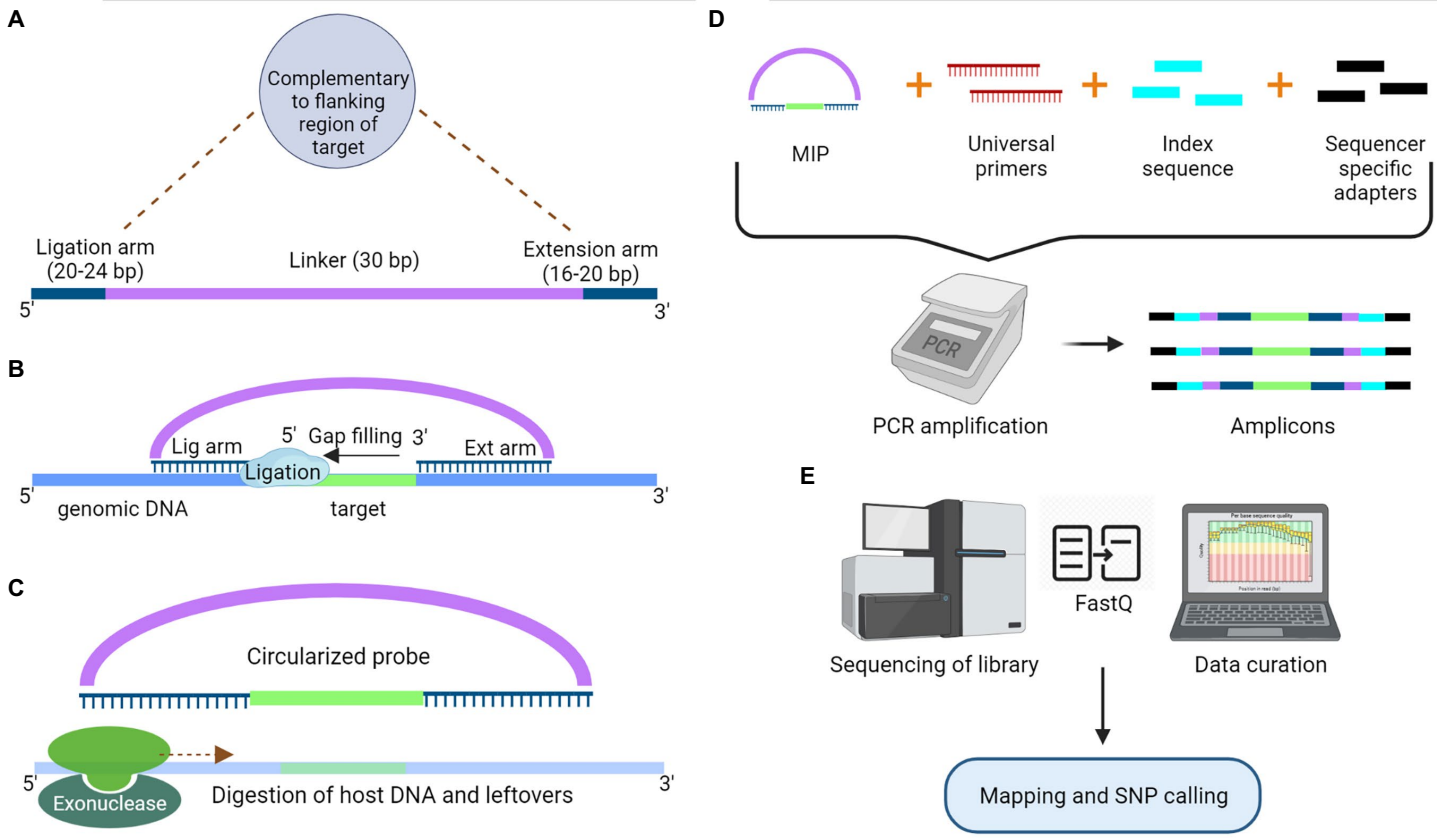
Schematic representation of Molecular Inversion Probe to capture a specific target for sequencing. **(A)** Model structure of a molecular inversion probe (MIP) with a ligation and extension arm connected by a 30 bp linker. Both ligation and extension arms are designed to complement the target region. **(B)** These complementary arms at the end of linker pairs with the target region, which is followed by gap filling and ligation (Circular DNA molecule). **(C)** Digestion of exogenous host DNA and probes with the help of exonucleases. **(D)** Amplification of the captured segments using the universal primers complementary to the linker sequence of MIP along with sample-specific index sequences and sequencer-specific adapters. **(E)** Libraries will go through the sequencing followed by data curation.

The target enrichment approach offers more specificity and replicable results than the RE based GBS approach (Malmberg et al., 2018). However, prior knowledge of genomic regions of interest is required to design suitable primers/probes for this approach. The most straightforward targeted sequencing protocol for enrichment of small sized genomic targets is the PCR-based method. Moreover, this method provides high efficiency when the targeted regions have highly conserved sequences across cultivars at the primer binding sites (Kaur and Gaikwad, 2019). Nowadays, multiplex PCR products are used to exploit the high-throughput nature of NGS; however, the primer design for a large genome is cumbersome, and amplification often requires specific commercial DNA polymerase enzymes capable of amplifying long genomic DNA (~30 kb) (Ostezan et al., 2021). Additionally, target enrichment can be achieved through hybrid capture by using oligonucleotide probes complementary to the genomic area of interest (Fu et al., 2010). It is also a technically demanding and time-consuming method, which requires library construction before hybridization. The efficiency of this method drops drastically when the targeted regions are small, and the increase in sample size makes the process more time-consuming (Niedzicka et al., 2016). The two methods based on PCR and hybrid capture were combined to develop a hybrid protocol known as molecular inversion probes (MIPs), overcoming the deficiency in both methods. MIPs are single-stranded DNA molecules containing sequences at both ends that are complementary to regions flanking the target sequence and linked by a highly repetitive sequence (linker sequence). First, the hybridization of MIP to the target sequence takes place, followed by gap filling with dNTPs and ligation to form a circular DNA molecule (Figure 3). This molecule is used as a template for PCR reaction using the primers complementary to the linker sequence. Apart from the primers, sequencer-specific adapters and index sequences are also introduced during the PCR process. Now, the amplicons were sequenced for SNP detection. MIPs can target genomic regions ranging in size from 1 kb to 5 Mb, and they are more suited for targeted resequencing of thousands of short genomic regions in species with even partial genomic-level information (Niedzicka et al., 2016). These targeted sequencing approaches based on PCR, hybrid capture and MIPs have limitations as they do not detect any novel variants outside the targeted region and fail to differentiate similar genomic regions (Deschamps et al., 2012; Winfield et al., 2012).

**Figure 4:**
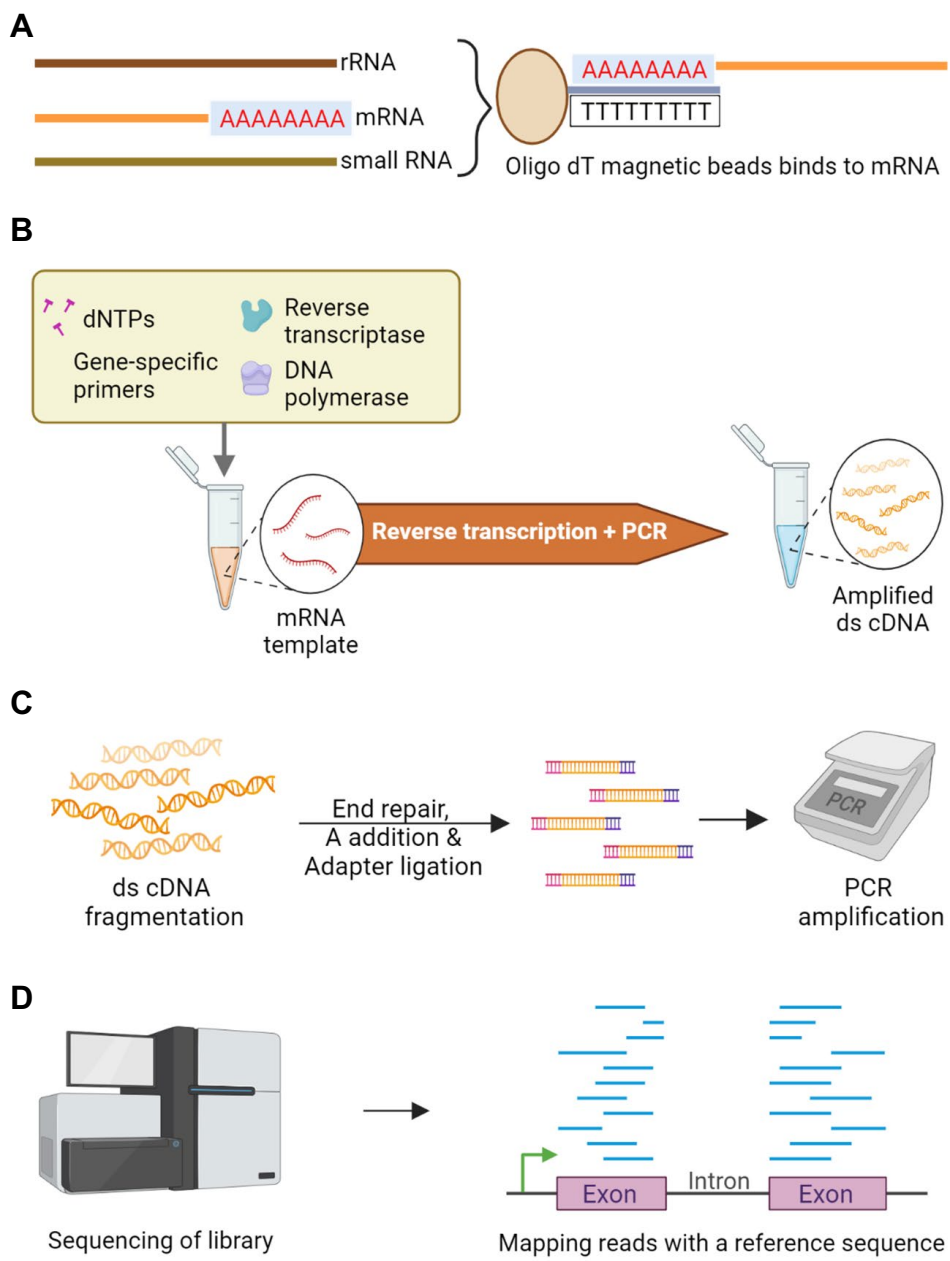
Schematic representation of RNA-Seq to sequence the transcriptome. **(A)** Poly A tailed messenger RNA (mRNA) is isolated among other RNA types using oligo dT magnetic beads. **(B)** Double-stranded complementary DNA (cDNA) library is constructed by employing reverse transcriptase on the isolated mRNA. **(C)** Enzymatic fragmentation of double-stranded cDNA is carried out to construct uniform library followed by end repair and an A nucleotide is added-to facilitate adapter ligation. These fragments are subjected to PCR amplification. **(D)** The amplicons are sequenced and differentially expressed genes are identified by mapping the reads with a quality reference sequence comparing the contrasting genotypes.

**Figure 5:**
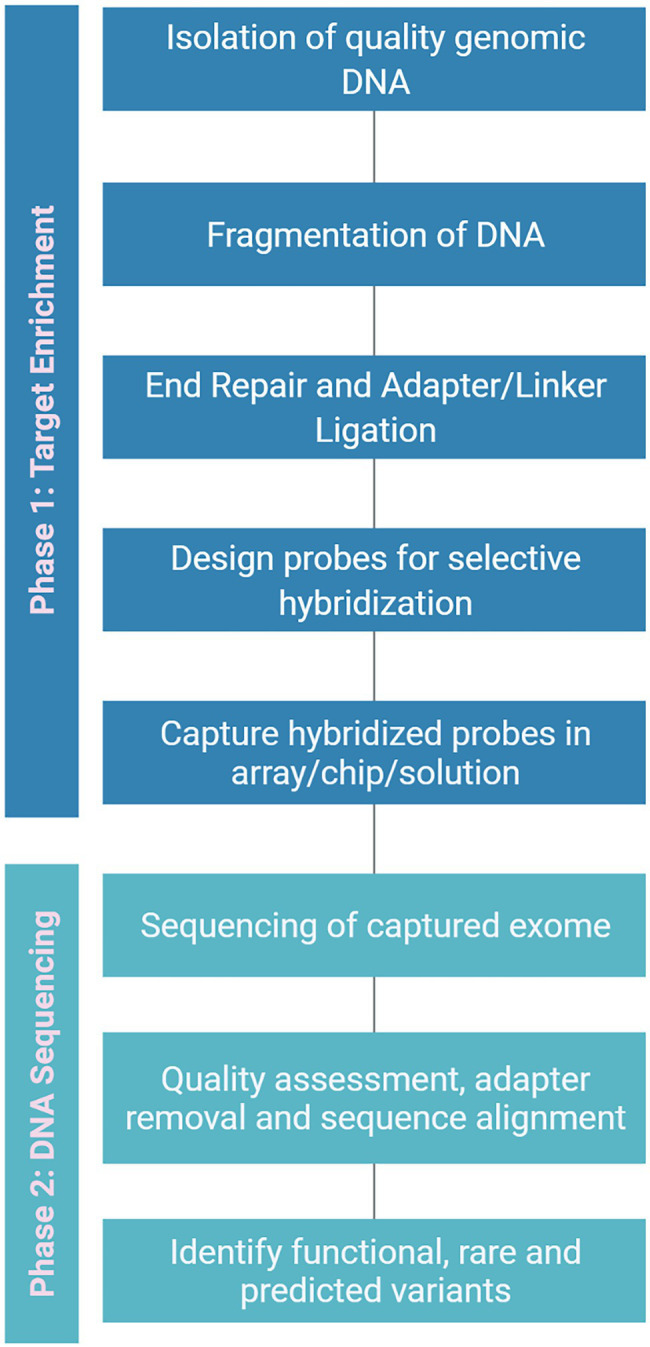
A generalized flowchart for exome capture to isolate the exon variants. It can be broadly divided into two phases *viz*., target enrichment and DNA sequencing. In enrichment phase high-quality genomic DNA is isolated and specific probes are designed for hybridization followed by capturing of hybridized probes. In sequencing phase, the raw exome sequence data is filtered and aligned to find potential variants.

RNA-Seq capturing the whole transcriptome, is another targeted sequencing approach that can reliably identify genetic variations in genic regions (Figure 4). It has been a reliable method for evaluating the potential genome and transcriptome level polymorphisms for genome-wide association studies. The major advantage of RNA-Seq is that no pre-requisite knowledge of genome sequence is required. However, the over-representation of abundant transcripts and challenges in the detection of sequences with low expression level remains a roadblock while assembling gene expression profiles (Ostezan et al., 2021), and splice variants can also sometimes add to the complexity. The assembly of these transcripts can be improved by adopting targeted RNA-Seq, which focuses only on the amplification of the genes of interest by designing probes complementary to the targeted exons (Levin et al., 2009; Mercer et al., 2014). This could also reduce the cost as there is no need for whole transcriptome sequencing (Arts et al., 2017). The whole exome capture (Figure 5) approach, using probes designed from target genic regions to hybridize genomic DNA, resolved the limitations related to the proportion of transcripts present or the crop’s developmental stage in RNA-Seq based approach (Kaur and Gaikwad, 2017). The selective sequencing of exome can be performed by three approaches: PCR-based capture, hybrid capture and array-based capture. One well-known example of selective exome capture is about a specific gene family with a nucleotide binding site/leucine-rich repeat (NLR) domain that triggers signaling in plants during pathogen invasion. NLR baits were designed to study this gene family and its molecular role during the plant-microbe interaction (Dinh et al., 2020).

**Figure 6:**
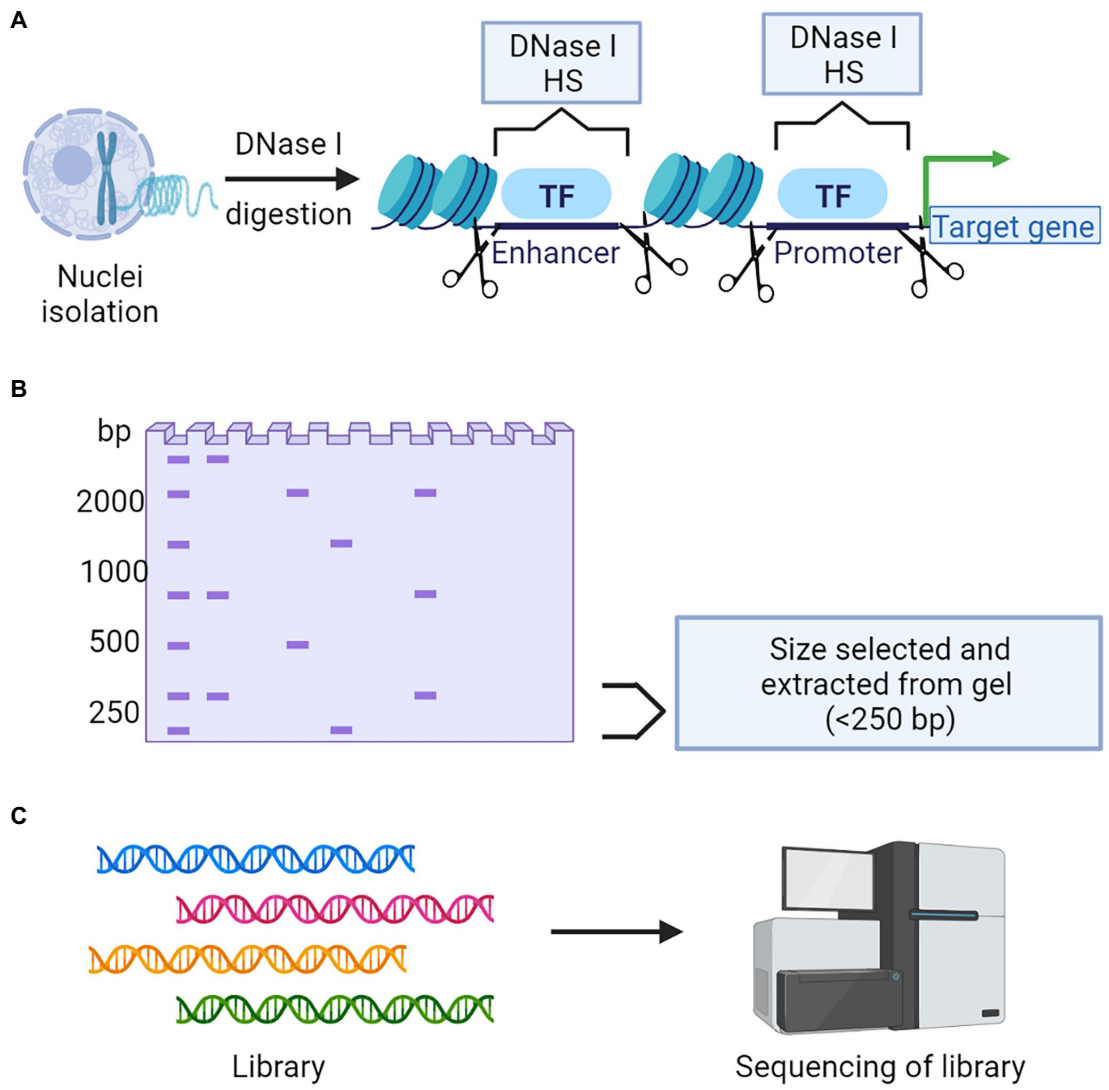
A schematic representation of DNase Seq to determine chromatin accessibility. **(A)** Nuclei isolation followed by DNase I digestion is a critical step to collect the chromatin. **(B)** Targeted fragments will be selected and isolated from gel. **(C)** Library construction followed by next-generation sequencing and data analysis.

**Figure 7:**
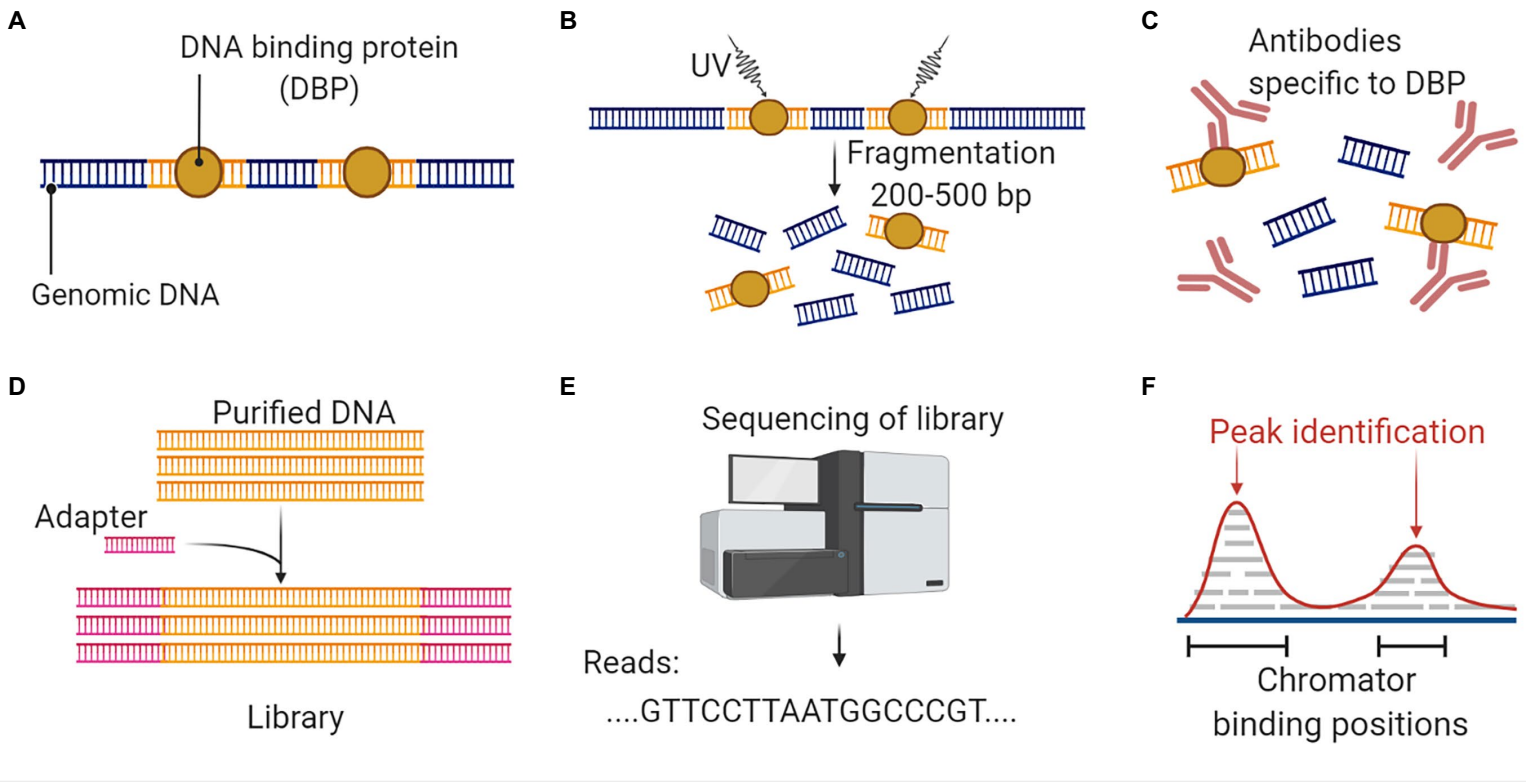
An overall workflow for ChIP Seq to analyze protein interactions with DNA. **(A)** Isolation of genomic DNA with conserved binding proteins. **(B)** Crosslinking and DNA fragmentation to access the DNA-protein complex. **(C)** Immunoprecipitation with protein-specific antibodies to separate the DNA-protein complex. **(D)** DNA purification is followed by adaptor ligation to prepare the sequencing library. **(E)** Discovery of DNA biding segments using next-generation sequencing platform. **(F)** Data analysis and alignment to reference genome will identify targeted DNA sequences that interact with the protein. This figure is adapted from “ChIP sequencing,” by BioRender.com (2022). Retrieved from https://app.biorender.com/biorender-templates.

DNase I hypersensitive sites (DHSs) in the genome usually harbors cis-regulatory DNA elements (CREs). CREs interact with transcription factors and guide the transcription process (Wang and Wang, 2021). DHSs are sensitive to the endonuclease DNase I, which can cleave these regions. The digested segments are used for sequencing to identify the important CREs and TFs (Mathelier et al., 2015). DHS mapping combined with NGS is popularly known as DNase-Seq (Figure 6). Another targeted region sequencing approach, which has regulatory importance is the chromatin immunoprecipitation sequencing (ChIP-Seq) that identifies the binding sites of DNA associated proteins. It refers to mapping of the binding proteins and their bio-chemical modifications across the genome, which play a vital role in replication, transcription, RNA processing, and DNA repair mechanisms (Furey, 2012). The unique feature of this protocol is that it involves the initial crosslinking step to fix the protein-DNA binding *in vivo*, allowing the capture of native interactions. Antibodies specific to the protein of interest was used to immuno-precipitate the crosslinked DNA and protein. The extracted fragments were sequenced, and the resulting reads were mapped to the genome (Figure 7).

## SNP Variant Calling

The analysis of GBS sequence data can be complex; hence, there is a need for bioinformatic pipelines with advanced computational efficiency to sort, classify based on sequence barcodes, filter out poor-quality reads, score individuals based on their genotypes, and align the sequenced reads to the given reference sequence for SNP variant calling (Torkamaneh et al., 2016).

Bioinformatic pipelines for SNP variant calling are mainly categorized into two groups: with or without a reference genome. The commonly used pipelines which require a reference genome for data analysis are TASSEL-GBS (v.1 and v.2), IGST, and Fast-GBS, whereas the pipeline that does not require a reference genome is UNEAK (Bradbury et al., 2007; Lu et al., 2013; Sonah et al., 2013; Glaubitz et al., 2014; Torkamaneh et al., 2017). The pipeline Stacks can be used with or without a reference genome for SNP detection (Catchen et al., 2013), although the confidence of allele calling varies. The UNEAK and Stacks pipelines would identify pairs of nearly identical reads, which could potentially represent alternative alleles at a single locus (Catchen et al., 2013; Lu et al., 2013). The TASSEL-GBS or Fast-GBS can map sequencing reads onto the reference genome to identify SNPs, which is a more robust approach (Li and Durbin, 2009; Nielsen et al., 2011). The imputation procedure of missing data generated from GBS is more accurate when a reference sequence is present (Torkamaneh and Belzile, 2015). Therefore, TASSEL-GBS has been widely utilized in association analysis in barley for which the reference genome is available (Pasam et al., 2012; Scheben et al., 2017). Also, it can handle large volumes of low coverage data (Scheben et al., 2017). Although, TASSEL-GBS has been used in many studies with barley to generate SNPs, the alternative Fast-GBS is emerging as a more powerful pipeline providing more thorough data analysis. The efficiency of Fast-GBS was demonstrated in soybean lines by comparing with other pipelines and sequencing platforms. The study found that the SNP calls from a single sequencing technology using different pipelines (Fast-GBS, Stacks, UNEAK, TASSEL-GBS, IGST) had a common SNP overlapping percentage ranging from 72 to 92%, whereas very low overlapping percentage ranging from 50 to 70% was observed while using a single pipeline with the data generated from two different sequencing technologies (Illumina and Ion Torrent). Such variations in overlapping percentage occur due to different variant calling algorithms and read mapping in each pipeline (O’Rawe et al., 2013). Fast-GBS is more flexible and accurate than other pipelines such as, TASSEL-GBS v.2 and Stacks, as demonstrated in soybean (Torkamaneh et al., 2016). In Fast-GBS, various data filtering parameters such as, quality scores for reads, number of reads required, and missing data allowed, can be altered based on the requirements of the study, irrespective of the sequencing platform used. This flexibility makes Fast-GBS less prone to errors. In barley, Fast-GBS mined out more SNPs than the UNEAK pipeline and it has proved to be far more user-friendly (Abed et al., 2019). In addition to these pipelines, researchers have also been using several inhouse bioinformatic pipelines using different programming languages.

Irrespective of the pipelines used, it is necessary to always perform additional quality checks and filtration on datasets (Abed et al., 2019). A well-defined protocol for filtering high-quality data using variant call format (VCF) tools has been demonstrated in crops, including barley (Danecek et al., 2011; Abed et al., 2019). For filtering, parameters such as, heterozygosity, sequencing depth, SNP quality, call rate, missing data percentage and minor allele frequency range (false SNPs) are usually preferred. In order to obtain a complete SNP catalogue, missing genotypic data can be inferred by imputation (Abed et al., 2019) or it can be excluded from downstream genetic analysis (Nazzicari et al., 2016). For data imputation, several software programs are available, including BEAGLE (Browning and Browning, 2007), FImpute (Sargolzaei et al., 2014), and LinkImpute (Money et al., 2015). BEAGLE and FImpute are the most commonly used packages, which exploit the linkage disequilibrium, haplotype information, and pedigree relationships to achieve accurate imputation of missing data (Shi et al., 2017). LinkImpute is optimized for the imputation of missing data for unrelated germplasm; thus, selection of the statistical software package for imputation is based on the objective of the study and the type of germplasm used. The fastPHASE package was used in barley to impute missing data of a low-density experimental panel based on data from a high-density reference panel (Iwata and Jannink, 2010). Data imputation is only optional, and any necessity to do it only depends on the research objectives.

## Applications of GBS in Barley

GBS technologies have allowed breeders to access a greater range of genetic variations to construct genetic maps, conduct linkage and genome-wide association studies (GWAS), execute genomic selection (GS), and assess genetic diversity in germplasm (Table 2).

**Table 2:** Practical application of GBS in barley.

S/No.	Population name	Population structure	GBS protocol	RE	Sequencing platform	Reference panel	Key findings	References
1	Commander × Fleet, Commander × WI4304, Fleet × WI4304	DHs	One enzyme	*Ape*KI	Illumina Hiseq 2000	IBSC barley reference sequence v.1	Identified 18 QTL for yield (1H, 2H, 4H, 6H, 7H) and 17 QTL for grain plumpness (1H-7H)	Obsa et al., 2017
2	Chevallier × NFC Tipple	RILs	One enzyme	*Ape*KI	Illumina Beadxpress	Beadxpress cultivar optimized SNP (384) panel	Identified QTL for plant height, reduced tiller number, susceptibility to PM (1H) & a novel QTL for physiological leaf spotting (3H, 7H)	Goddard et al., 2019
3	Golden promise × Morex	RILs	Two enzyme	*Pst*I, *Mse*I	Illumina GA II & Illumina Hiseq 2000	Genome assembly of Morex, Bowman & Barke cv.	Identified 3 QTL for plant height (2H, 3H & 5H)	Liu et al., 2014
4	*Hordeum vulgare* L. cv. Borwina × *Hordeum bulbosum* L.	Hb introgression lines	Two enzyme	*Pst*I-HF, *Msp*I	Illumina Hiseq 2000	Barley Reference sequence (IBSC)	Identified and mapped introgressed segments of Hb on Hv	Wendler et al., 2014
5	HOR2573 × Morex	RILs	Two enzyme	*Pst*I, *Msp*I	Illumina Hiseq 2,500	Barley reference genome	Identified four powdery mildew resistant candidate genes (2H)	Hoseinzadeh et al., 2019
6	Barley advanced lines & varieties	-	Two enzyme	*Pst*I, *Msp*I	Two Ion PI chips	IBSC barley reference sequence v.2	Suggested an integrated GWAS (single-SNP, multiple SNP & Haplotype) approach for precise QTL detection	Abed and Belzile, 2019
7	IPK barley collection (coreset)	-	Two enzyme	*Pst*I, *Msp*I	Illumina Hiseq 2,500	Barley reference genome	Identified key regions for awn roughness (5H, 7H) & lateral spikelet fertility (1H)	Milner et al., 2019
8	*H. vulgare* L. cv. Borwina (Tetraploid) × *H. bulbosum* L.	Hb introgression lines	Two enzyme	*Pst*I, *Msp*I	Illumina Hiseq 2,500	TRITEX genome assembly (Morex)	Introgressed and located powdery mildew resistant locus (Mlhb.A42) from Hb to Hv (2HS)	Hoseinzadeh et al., 2020
9	Bowman × ND 5883	RILs	Two enzyme	*Pst*I, *Mse*I	BGI500	Morex genome pseudo molecules and contigs sequences	Identified 2 QTL for susceptibility to spot blotch (1H, 7H)	Leng et al., 2020
10	Nierumuzha × Kunlun 10	DHs	GBS pre-design by Novogene, China	*Hae*III, *Mse*I, *Eco*RI	Illumina Hiseq	Barley reference genome	Identified major loci for purple seed coat using mapping and gene annotation (4H, 7H)	Yao et al., 2018
11	Wild and Domesticated barley	–	–	–	–	Raw GBS reads from European Nucleotide Archives	Identified 532,253 pan-genome sequence anchors based on the presence/absence tags	Gao et al., 2020

### Genetic Mapping and QTL Detection

GBS platform has enhanced the ability to produce high-density molecular maps in cereals, a pre-requisite for trait-marker linkage studies (Chung et al., 2017). The development of genetic materials and effective implementation of high-throughput phenotyping will remain the main challenges in using this genomic data for crop improvement. High-density molecular maps combined with precise phenotype from bi-parental and multi-parental populations, has allowed to uncover the genetic architecture underlying Mendelian traits and complex traits by genetic mapping analysis (Poland and Rife, 2012). For agronomic traits, GBS based genetic maps were generated in barley recombinant inbred lines to identify three QTLs for plant height on chromosome 2H, 3H and 5H, where the QTL on 2H was positioned near a locus conferring for spike architecture (*Vrs1*), and the QTL on 5H was in close proximity to a dwarfing gene locus (*Ari-e*) (Liu et al., 2014). Similarly, DH lines developed from reciprocal crosses involving three parents were utilized to identify 17 and 18 QTLs for grain plumpness and yield, respectively, under drought-prone environments (Obsa et al., 2017). With a high-density genetic map of 3,662 SNP markers generated by GBS approach, a total of five loci and a regulatory factor related to flavonoid synthesis were identified for the economically important purple seed coat trait (Yao et al., 2018).

For disease resistance, various GBS based genetic maps have successfully identified critical QTLs related to powdery mildew and spot blotch. A RIL population with a genetic map length of 1,000 cM was used to identify four candidate genes for powdery mildew resistance on chromosome 2H (Hoseinzadeh et al., 2019). In another study, a susceptible QTL on chromosome 1H for powdery mildew resistance was identified by GBS approach (Goddard et al., 2019). Two QTLs on chromosome 1H and 7H for spot blotch susceptibility were identified from a high-density map and the QTL on chromosome 1H (Qsbs-1H-PI) was found to be a novel allele (Leng et al., 2020).

The one enzyme GBS approach (Elshire et al., 2011) was used to develop high-density genetic maps for the DH population of Oregon Wolfe Barley and Morex × Barke. These genetic maps have been used as a genetic framework to develop the barley physical map, a reference assembly for various breeding and genetic research (Mayer et al., 2012). Similar reference quality anchoring of SNP related contigs was also carried out, which increased the genetically anchored contigs by three times (Mascher et al., 2013).

### Genome-Wide Association Studies

GWAS can provide high-resolution mapping by making use of multiple recombination events over many generations (Yu and Buckler, 2006). It is considered as one of the most powerful tools for identifying marker-trait associations using large populations. GWAS have high resolution due to the exploitation of historical recombination events that are limited in bi-parental populations (Zheng et al., 2008). One of the most significant setbacks in GWAS could be the need for large numbers of markers (in millions), depending on the extent of linkage disequilibrium, but GBS is well suited to provide the required high-density markers (Chung et al., 2017). However, for self-pollinated crops like barley and wheat, a few thousand SNPs are usually sufficient to cover the whole genome, given that they have large linkage disequilibrium blocks (Jighly et al., 2015). Another challenge for GWAS is the occurrence of false positives, especially when dealing with complex population structures. However, these false positives can be corrected to some extent by fitting the population structure as covariates using different models like the mixed linear model, which is capable of handling a single locus at a given time, whereas models such as, ISIS EM-BLASSO (Tamba et al., 2017), LASSO (Xu et al., 2017) and FarmCPU (Liu et al., 2016) are capable of handling multiple locus, simultaneously (Kaler and Purcell, 2019). Despite these challenges, GWAS remains a powerful method to understand the genetic architecture of traits.

Plant Genetics and Crop Plant Research institute in Germany (IPK) applied a two enzyme GBS approach to identify genomic regions conferring row type, hull adherence, awn roughness on a barley core collection. With the GWAS approach, QTL for awn roughness and lateral spikelet fertility were identified precisely on chromosome 1H, 5H and 7H (Milner et al., 2019). A study was conducted using GBS data from advanced barley lines and found that adopting an integrated approach involving both single and multi-locus GWAS had a better performance while detecting QTLs for complex traits (Abed and Belzile, 2019).

### Genomic Selection

Genomic selection (GS) is a rapidly evolving approach in breeding to predict the genetic value of individuals for selection. This technique was developed to tackle the challenges occurring in both linkage mapping and GWAS while dealing with complex traits particularly governed by minor QTLs (Srivastava et al., 2020) and traits showing high genotype by environment interaction (Jighly et al., 2021). In other words, GS is a more comprehensive version of marker-assisted selection (MAS), where DNA markers covering the whole genome are utilized to select superior genotypes. Once the genomic selection model is established, genotypic data can be used to select for or against lines without phenotyping (Heffner et al., 2009). In GS, a prediction model is developed by using both phenotypic and genotypic information collected from a training or reference population, and then this model is validated by independent testing populations. The double-checked trained population is utilized to formulate a standardized genomic estimated breeding value (GEBV), estimated as the sum of all the genotyped marker effects. The standard GEBV value of the trained model can be used to calculate the GEBV of an untrained population for selection without phenotyping (Contaldi et al., 2021). The high-density, genome-wide data achieved by GBS method is well suited to calculate the sum of marker effects. The genetic maps constructed using GBS provide a guide for the genomic selection of economically important traits and expand the capacity to detect genetic loci with minor effects on a phenotype. Raw GBS reads were retrieved from the European Nucleotide Archive, and 532,253 pan-genome sequence anchors were identified based on the presence/absence of tags. These tags are essential to identify desirable genomic regions within in a species and this information can be effectively used in genomic selection and improvement of barley (Gao et al., 2020).

### Diversity Studies

Analysis of DNA sequence variation between individuals is the most efficient way to study the genetic distance among them. This study allows comparisons across different species, or different lines of the same subspecies. Genetic diversity studies based on GBS simplify the complex alignment and related computational challenges that researchers often encounter while working on species with high genetic diversity (Elshire et al., 2011). The genetic relationship and geographical distribution of 16 diverse barley landraces were analyzed using the Roche 454 GS FLX titanium technology and GBS. The application of NGS has provided new information on the available barley genomic resources and revealed the diversity present between the barley landraces from both eastern (Zagros mountains and further east) and western (Fertile Crescent and further west) regions (Fu and Peterson, 2011). In another study, GBS data derived from 21,405 accessions from IPK collection were utilized to understand the population structure of domesticated barley and selected a core set of 1,000 genotypes (Milner et al., 2019). The same core set was used to evaluate the efficiency and effectiveness of both GBS and SNP array platforms. Both platforms were equally good in detecting informative SNPs; however, GBS held an edge over SNP array for the detection of rare alleles in the germplasm collection (Darrier et al., 2019).

## Conclusion

In the ever-evolving field of genomics, GBS platforms have taken a special place among other sequencing techniques to deliver high-density molecular maps, which are the essential pre-requisite for many advanced breeding studies, such as GWAS and GS, at reduced cost. The concept of genome reduction is the central principle underlying the wider acceptance of GBS, which is highly applicable in crops with large complex genomes, including wheat and barley. GBS works well with crops without any prior reference sequence information, but the availability of a high-quality reference sequence would increase the accuracy manifolds during sequence data curation and allow access to a higher proportion of sequence reads. In the future, standardization of multiple combinations of REs to construct libraries will provide opportunities to increase sequencing coverage. With regard to data analysis, GBS data generated from the existing protocols can be used in the future with the evolution of the computing power of GBS pipelines. The target enrichment based GBS methods have also gained traction; however, the technical complexity associated with these techniques could act as a hurdle in their wider adoption. Moreover, in barley, robust QTL, GWAS and GS studies have been conducted using the data generated from various platforms including GBS; nevertheless, meta-analysis presenting results by combining data from these individual studies are still very limited and need to be extended to encompass a broader scope in coming years. In the wake of climate change, it is now imperative to overcome the bottlenecks in barley breeding by exploiting every advance made in the post-NGS era.

## Author Contributions

NR, NQ, and MP contributed to the conception and design of the manuscript. NR wrote the manuscript drafts. NQ and MP revised the manuscript and compiled a final draft. All authors listed have made a substantial, direct, and intellectual contribution to the work and approved it for publication.

## Funding

This work was supported by the School of Agriculture and Food at the University of Melbourne to MP.

## Conflict of Interest

The authors declare that the research was conducted in the absence of any commercial or financial relationships that could be construed as a potential conflict of interest.

## Publisher’s Note

All claims expressed in this article are solely those of the authors and do not necessarily represent those of their affiliated organizations, or those of the publisher, the editors and the reviewers. Any product that may be evaluated in this article, or claim that may be made by its manufacturer, is not guaranteed or endorsed by the publisher.

## Footnotes

^1^ https://www.fao.org/wiews/data/domains/monitoring-framework/en/

^2^ www.genesys-pgr.org

^3^ https://seedvault.nordgen.org/Search

^4^ https://www.nordgen.org/bgs/

^5^ http://plants.ensembl.org/Hordeum_vulgare

^6^ https://ics.hutton.ac.uk/barleyGenes/

^7^ https://ics.hutton.ac.uk/eorna/index.html

## References

[ref1] Abed A. Belzile F. (2019). Comparing single-SNP, multi-SNP, and haplotype-based approaches in association studies for major traits in barley. Plant Genome 12:190036. doi: 10.3835/plantgenome2019.05.0036 PMC12810143 33016584

[ref2] Abed A. Légaré G. Pomerleau S. St-Cyr J. Boyle B. Belzile F. J. (2019). Genotyping-by-Sequencing on the Ion Torrent Platform in Barley. New York: Springer New York, 233–252. 10.1007/978-1-4939-8944-7_15 30460569

[ref3] Alqudah A. M. Sallam A. Stephen Baenziger P. Börner A. (2020). GWAS: fast-forwarding gene identification and characterization in temperate cereals: lessons from barley – a review. J. Adv. Res. 22, 119–135. doi: 10.1016/j.jare.2019.10.013 31956447 PMC6961222

[ref4] Arts P. Van Der Raadt J. Van Gestel S. H. C. Steehouwer M. Shendure J. Hoischen A.. (2017). Quantification of differential gene expression by multiplexed targeted resequencing of cDNA. Nat. Commun. 8:15190. doi: 10.1038/ncomms15190 28474677 PMC5424154

[ref5] Babu R. Nair S. K. Prasanna B. M. Gupta H. S. (2004). Integrating marker-assisted selection in crop breeding – prospects and challenges. Curr. Sci. 87, 607–619.

[ref6] Baird N. A. Etter P. D. Atwood T. S. Currey M. C. Shiver A. L. Lewis Z. A.. (2008). Rapid SNP discovery and genetic mapping using sequenced RAD markers. PLoS One 3:e3376. doi: 10.1371/journal.pone.0003376 18852878 PMC2557064

[ref7] Bajgain P. Rouse M. N. Anderson J. A. (2016). Comparing genotyping-by-sequencing and single nucleotide polymorphism chip genotyping for quantitative trait loci mapping in wheat. Crop Sci. 56, 232–248. doi: 10.2135/cropsci2015.06.0389

[ref8] Bayer M. M. Rapazote-Flores P. Ganal M. Hedley P. E. Macaulay M. Plieske J.. (2017). Development and evaluation of a barley 50k iSelect SNP array. Front. Plant Sci. 8:1792. doi: 10.3389/fpls.2017.01792 29089957 PMC5651081

[ref9] Beissinger T. M. Hirsch C. N. Sekhon R. S. Foerster J. M. Johnson J. M. Muttoni G.. (2013). Marker density and read depth for genotyping populations using genotyping-by-sequencing. Genetics 193, 1073–1081. doi: 10.1534/genetics.112.147710 23410831 PMC3606087

[ref10] Bentley D. R. Balasubramanian S. Swerdlow H. P. Smith G. P. Milton J. Brown C. G.. (2008). Accurate whole human genome sequencing using reversible terminator chemistry. Nature 456, 53–59. doi: 10.1038/nature07517 18987734 PMC2581791

[ref11] Bhat J. A. Ali S. Salgotra R. K. Mir Z. A. Dutta S. Jadon V.. (2016). Genomic selection in the era of next generation sequencing for complex traits in plant breeding. Front. Genet. 7:221. doi: 10.3389/fgene.2016.00221 28083016 PMC5186759

[ref12] Bhavani S. Singh P. K. Qureshi N. He X. Biswal A. K. Juliana P.. (2021). Globally Important Wheat Diseases: Status, Challenges, Breeding and Genomic Tools to Enhance Resistance Durability. Berlin: Springer International Publishing, 59–128.

[ref13] Bradbury P. J. Zhang Z. Kroon D. E. Casstevens T. M. Ramdoss Y. Buckler E. S. (2007). TASSEL: software for association mapping of complex traits in diverse samples. Bioinformatics 23, 2633–2635. doi: 10.1093/bioinformatics/btm308 17586829

[ref14] Browning S. R. Browning B. L. (2007). Rapid and accurate haplotype phasing and missing-data inference for whole-genome association studies by use of localized haplotype clustering. Am. J. Hum. Genet. 81, 1084–1097. doi: 10.1086/521987 17924348 PMC2265661

[ref15] Catchen J. Hohenlohe P. A. Bassham S. Amores A. Cresko W. A. (2013). Stacks: an analysis tool set for population genomics. Mol. Ecol. 22, 3124–3140. doi: 10.1111/mec.12354, PMID: 23701397 PMC3936987

[ref16] Chung Y. S. Choi S. C. Jun T.-H. Kim C. (2017). Genotyping-by-sequencing: a promising tool for plant genetics research and breeding. Hortic. Environ. Biotechnol. 58, 425–431. doi: 10.1007/s13580-017-0297-8

[ref17] Close T. J. Bhat P. R. Lonardi S. Wu Y. Rostoks N. Ramsay L.. (2009). Development and implementation of high-throughput SNP genotyping in barley. BMC Genomics 10:582. doi: 10.1186/1471-2164-10-582 19961604 PMC2797026

[ref18] Comadran J. Kilian B. Russell J. Ramsay L. Stein N. Ganal M.. (2012). Natural variation in a homolog of Antirrhinum CENTRORADIALIS contributed to spring growth habit and environmental adaptation in cultivated barley. Nat. Genet. 44, 1388–1392. doi: 10.1038/ng.2447 23160098

[ref19] Contaldi F. Cappetta E. Esposito S. (2021). Practical Workflow from High-Throughput Genotyping to Genomic Estimated Breeding Values (GEBVs). New York: Springer, US. 119–135. 10.1007/978-1-0716-1201-9_9 33263907

[ref20] Craig D. W. Pearson J. V. Szelinger S. Sekar A. Redman M. Corneveaux J. J.. (2008). Identification of genetic variants using bar-coded multiplexed sequencing. Nat. Methods 5, 887–893. doi: 10.1038/nmeth.1251 18794863 PMC3171277

[ref21] Dacosta J. M. Sorenson M. D. (2014). Amplification biases and consistent recovery of loci in a double-digest RAD-seq protocol. PLoS One 9:e106713. doi: 10.1371/journal.pone.0106713 25188270 PMC4154734

[ref22] Danecek P. Auton A. Abecasis G. Albers C. A. Banks E. Depristo M. A.. (2011). The variant call format and VCFtools. Bioinformatics 27, 2156–2158. doi: 10.1093/bioinformatics/btr330 21653522 PMC3137218

[ref23] Darrier B. Russell J. Milner S. G. Hedley P. E. Shaw P. D. Macaulay M.. (2019). A comparison of mainstream genotyping platforms for the evaluation and use of barley genetic resources. Front. Plant Sci. 10:544. doi: 10.3389/fpls.2019.00544 31105733 PMC6499090

[ref25] Deschamps S. Llaca V. May G. D. (2012). Genotyping-by-sequencing in plants. Biology 1, 460–483. doi: 10.3390/biology1030460 24832503 PMC4009820

[ref26] Dinh H. X. Singh D. Periyannan S. Park R. F. Pourkheirandish M. (2020). Molecular genetics of leaf rust resistance in wheat and barley. Theor. Appl. Genet. 133, 2035–2050. doi: 10.1007/s00122-020-03570-8 32128617

[ref27] Dou J. Zhao X. Fu X. Jiao W. Wang N. Zhang L.. (2012). Reference-free SNP calling: improved accuracy by preventing incorrect calls from repetitive genomic regions. Biol. Direct 7:17. doi: 10.1186/1745-6150-7-17, PMID: 22682067 PMC3472322

[ref29] Elshire R. J. Glaubitz J. C. Sun Q. Poland J. A. Kawamoto K. Buckler E. S.. (2011). A robust, simple genotyping-by-sequencing (GBS) approach for high diversity species. PLoS One 6:e19379. doi: 10.1371/journal.pone.0019379 21573248 PMC3087801

[ref30] FAO (2010). The Second Report on State of the World’s Plant Genetic Resources for Food and Agriculture. FAO: Rome.

[ref1010] FAO WIEWS (2022). Ex situ search. Available at: https://www.fao.org/wiews/data/ex-situ-sdg-251/search/en/?no_cache=1 (Accessed February 27, 2022).

[ref31] Fellers J. P. (2008). Genome filtering using methylation-sensitive restriction enzymes with six base pair recognition sites. Plant Genome 1, 146–152. doi: 10.3835/plantgenome2008.05.0245

[ref32] Fu Y.-B. Peterson G. W. (2011). Genetic diversity analysis with 454 pyrosequencing and genomic reduction confirmed the eastern and western division in the cultivated barley gene pool. Plant Genome 4, 226–237. doi: 10.3835/plantgenome2011.08.0022

[ref33] Fu Y. Springer N. M. Gerhardt D. J. Ying K. Yeh C.-T. Wu W.. (2010). Repeat subtraction-mediated sequence capture from a complex genome. Plant J. 62, 898–909. doi: 10.1111/j.1365-313x.2010.04196.x 20230488

[ref34] Furey T. S. (2012). ChIP–seq and beyond: new and improved methodologies to detect and characterize protein–DNA interactions. Nat. Rev. Genet. 13, 840–852. doi: 10.1038/nrg3306 23090257 PMC3591838

[ref35] Galluzzi G. Seyoum A. Halewood M. López Noriega I. Welch E. W. (2020). The role of genetic resources in breeding for climate change: the case of public breeding programmes in eighteen developing countries. Plan. Theory 9:1129. doi: 10.3390/plants9091129, PMID: 32878309 PMC7569780

[ref36] Gao S. Wu J. Stiller J. Zheng Z. Zhou M. Wang Y.-G.. (2020). Identifying barley pan-genome sequence anchors using genetic mapping and machine learning. Theor. Appl. Genet. 133, 2535–2544. doi: 10.1007/s00122-020-03615-y 32448920

[ref37] Garrido-Cardenas J. A. Mesa-Valle C. Manzano-Agugliaro F. (2018). Trends in plant research using molecular markers. Planta 247, 543–557. doi: 10.1007/s00425-017-2829-y 29243155

[ref202] Genesys (2022). Global Portal on Plant Genetic Resources. Available at: http://www.genesys-pgr.org/ (Accessed March 5, 2022).

[ref38] Glaubitz J. C. Casstevens T. M. Lu F. Harriman J. Elshire R. J. Sun Q.. (2014). TASSEL-GBS: a high capacity genotyping by sequencing analysis pipeline. PLoS One 9:e90346. doi: 10.1371/journal.pone.0090346, PMID: 24587335 PMC3938676

[ref39] Gnirke A. Melnikov A. Maguire J. Rogov P. Leproust E. M. Brockman W.. (2009). Solution hybrid selection with ultra-long oligonucleotides for massively parallel targeted sequencing. Nat. Biotechnol. 27, 182–189. doi: 10.1038/nbt.1523 19182786 PMC2663421

[ref40] Goddard R. De Vos S. Steed A. Muhammed A. Thomas K. Griggs D.. (2019). Mapping of agronomic traits, disease resistance and malting quality in a wide cross of two-row barley cultivars. PLoS One 14:e0219042. doi: 10.1371/journal.pone.0219042 31314759 PMC6636724

[ref41] Gupta P. K. Rustgi S. Mir R. R. (2008). Array-based high-throughput DNA markers for crop improvement. Heredity 101, 5–18. doi: 10.1038/hdy.2008.35 18461083

[ref42] Hardenbol P. Yu F. Belmont J. Mackenzie J. Bruckner C. Brundage T.. (2005). Highly multiplexed molecular inversion probe genotyping: over 10,000 targeted SNPs genotyped in a single tube assay. Genome Res. 15, 269–275. doi: 10.1101/gr.3185605 15687290 PMC546528

[ref43] Harwood W. A. (2019). An Introduction to Barley: The Crop and the Model. Springer: New York, 1–5. 10.1007/978-1-4939-8944-7_1 30460555

[ref44] Hasan N. Choudhary S. Naaz N. Sharma N. Laskar R. A. (2021). Recent advancements in molecular marker-assisted selection and applications in plant breeding programmes. J. Genetic Eng. Biotechnol 19:128. doi: 10.1186/s43141-021-00231-1 PMC8397809 34448979

[ref45] He J. Zhao X. Laroche A. Lu Z.-X. Liu H. Li Z. (2014). Genotyping-by-sequencing (GBS), an ultimate marker-assisted selection (MAS) tool to accelerate plant breeding. Front. Plant Sci. 5:484. doi: 10.3389/fpls.2014.00484 25324846 PMC4179701

[ref46] Heffner E. L. Sorrells M. E. Jannink J.-L. (2009). Genomic selection for crop improvement. Crop Sci. 49, 1–12. doi: 10.2135/cropsci2008.08.0512

[ref47] Hoseinzadeh P. Ruge-Wehling B. Schweizer P. Stein N. Pidon H. (2020). High resolution mapping of a *Hordeum bulbosum*-derived powdery mildew resistance locus in barley using distinct homologous introgression lines. *Front*. Plant Sci. 11:225. doi: 10.3389/fpls.2020.00225, PMID: 32194602 PMC7063055

[ref48] Hoseinzadeh P. Zhou R. Mascher M. Himmelbach A. Niks R. E. Schweizer P.. (2019). High resolution genetic and physical mapping of a major powdery mildew resistance locus in barley. Front. Plant Sci. 10:146. doi: 10.3389/fpls.2019.00146, PMID: 30838011 PMC6382739

[ref49] Huang X. Feng Q. Qian Q. Zhao Q. Wang L. Wang A.. (2009). High-throughput genotyping by whole-genome resequencing. Genome Res. 19, 1068–1076. doi: 10.1101/gr.089516.108 19420380 PMC2694477

[ref50] Iwata H. Jannink J.-L. (2010). Marker genotype imputation in a low-marker-density panel with a high-marker-density reference panel: accuracy evaluation in barley breeding lines. Crop Sci. 50, 1269–1278. doi: 10.2135/cropsci2009.08.0434

[ref51] Jayakodi M. Padmarasu S. Haberer G. Bonthala V. S. Gundlach H. Monat C.. (2020). The barley pan-genome reveals the hidden legacy of mutation breeding. Nature 588, 284–289. doi: 10.1038/s41586-020-2947-8, PMID: 33239781 PMC7759462

[ref52] Jighly A. (2022). When do autopolyploids need poly-sequencing data? Mol. Ecol. 31, 1021–1027. doi: 10.1111/mec.16313 34875138

[ref53] Jighly A. Hayden M. Daetwyler H. (2021). Integrating genomic selection with a genotype plus genotype x environment (GGE) model improves prediction accuracy and computational efficiency. Plant Cell Environ. 44, 3459–3470.doi: 10.1111/pce.14145 34231236

[ref54] Jighly A. Oyiga B. C. Makdis F. Nazari K. Youssef O. Tadesse W.. (2015). Genome-wide DArT and SNP scan for QTL associated with resistance to stripe rust (Puccinia striiformis f. sp. tritici) in elite ICARDA wheat (*Triticum aestivum* L.) germplasm. Theor. Appl. Genet. 128, 1277–1295. doi: 10.1007/s00122-015-2504-2 25851000

[ref55] Kaler A. S. Purcell L. C. (2019). Estimation of a significance threshold for genome-wide association studies. BMC Genomics 20:618. doi: 10.1186/s12864-019-5992-7 31357925 PMC6664749

[ref56] Kamal N. Lux T. Jayakodi M. Haberer G. Gundlach H. Mayer K. F. X.. (2022). The Barley and Wheat Pan-Genomes. New York: Springer US, 147–159. 10.1007/978-1-0716-2067-0_7 35037204

[ref57] Kant L. Amrapali S. Babu B. K. (2016). “Barley,” in Genetic and Genomic Resources for Grain Cereals Improvement. eds. Singh M. Upadhyaya H. D. (Cambridge, United Kingdom: Academic Press), 125–157.

[ref58] Kaur P. Gaikwad K. (2017). From genomes to GENE-omes: exome sequencing concept and applications in crop improvement. Front. Plant Sci. 8:2164. doi: 10.3389/fpls.2017.02164, PMID: 29312405 PMC5742236

[ref59] Kaur P. Gaikwad K. (2019). Principles and Implications of Various Genome Enrichment Approaches for Targeted Sequencing of Plant Genomes. Singapore: Springer Singapore, 43–75.

[ref60] Kim D. Paggi J. M. Park C. Bennett C. Salzberg S. L. (2019). Graph-based genome alignment and genotyping with HISAT2 and HISAT-genotype. Nat. Biotechnol. 37, 907–915. doi: 10.1038/s41587-019-0201-4 31375807 PMC7605509

[ref61] Kleinhofs A. Kilian A. Saghai Maroof M. A. Biyashev R. M. Hayes P. Chen F. Q.. (1993). A molecular, isozyme and morphological map of the barley (*Hordeum vulgare*) genome. Theor. Appl. Genet. 86, 705–712. doi: 10.1007/bf00222660 24193780

[ref62] Knüpffer H. (2009). “Triticeae Genetic Resources in ex situ Genebank Collections,” in Genetics and Genomics of the Triticeae. Plant Genetics and Genomics: Crops and Models. Vol. 7. eds. Muehlbauer G. Feuillet C. (New York: Springer US), 31–79.

[ref63] Komatsuda T. Pourkheirandish M. He C. Azhaguvel P. Kanamori H. Perovic D.. (2007). Six-rowed barley originated from a mutation in a homeodomain-leucine zipper I-class homeobox gene. Proc. Natl. Acad. Sci. 104, 1424–1429. doi: 10.1073/pnas.0608580104 17220272 PMC1783110

[ref64] Leng Y. Zhao M. Fiedler J. Dreiseitl A. Chao S. Li X.. (2020). Molecular mapping of loci conferring susceptibility to spot blotch and resistance to powdery mildew in barley using the sequencing-based genotyping approach. Phytopathology 110, 440–446. doi: 10.1094/phyto-08-19-0292-r 31609681

[ref65] Levin J. Z. Berger M. F. Adiconis X. Rogov P. Melnikov A. Fennell T.. (2009). Targeted next-generation sequencing of a cancer transcriptome enhances detection of sequence variants and novel fusion transcripts. Genome Biol. 10:R115. doi: 10.1186/gb-2009-10-10-r115 19835606 PMC2784330

[ref03] Li H. Durbin R. (2009). Fast and accurate short read alignment with Burrows–Wheeler transform. Bioinformatics 25, 1754–1760. doi: 10.1093/bioinformatics/btp324 19451168 PMC2705234

[ref66] Li C. Chen G. Mishina K. Yamaji N. Ma J. F. Yukuhiro F.. (2017). A GDSL −motif esterase/acyltransferase/lipase is responsible for leaf water retention in barley. Plant Direct 1:e00025. doi: 10.1002/pld3.25 31245672 PMC6508521

[ref67] Li C. Wang A. Ma X. Pourkheirandish M. Sakuma S. Wang N.. (2013). An eceriferum locus, cer-zv, is associated with a defect in cutin responsible for water retention in barley (*Hordeum vulgare*) leaves. Theor. Appl. Genet. 126, 637–646. doi: 10.1007/s00122-012-2007-3 23124432

[ref68] Liu H. Bayer M. Druka A. Russell J. R. Hackett C. A. Poland J.. (2014). An evaluation of genotyping by sequencing (GBS) to map the Breviaristatum-e (ari-e) locus in cultivated barley. BMC Genomics 15:104. doi: 10.1186/1471-2164-15-104, PMID: 24498911 PMC3922333

[ref69] Liu X. Huang M. Fan B. Buckler E. S. Zhang Z. (2016). Iterative usage of fixed and random effect models for powerful and efficient genome-wide association studies. PLoS Genet. 12:e1005767. doi: 10.1371/journal.pgen.1005767 26828793 PMC4734661

[ref70] Lu F. Lipka A. E. Glaubitz J. Elshire R. Cherney J. H. Casler M. D.. (2013). Switchgrass genomic diversity, ploidy, and evolution: novel insights from a network-based SNP discovery protocol. PLoS Genet. 9:e1003215. doi: 10.1371/journal.pgen.1003215 23349638 PMC3547862

[ref71] Lu F. Romay M. C. Glaubitz J. C. Bradbury P. J. Elshire R. J. Wang T.. (2015). High-resolution genetic mapping of maize pan-genome sequence anchors. Nat. Commun. 6:6914. doi: 10.1038/ncomms7914 25881062 PMC4411285

[ref72] Lundqvist U. Franckowiak J. D. (2003). “Diversity in barley mutants,” in Diversity in Barley (Hordeum vulgare L.). eds. von Bothmer R. van Hintum T. Knüpffer T. H. Sato K. Amsterdam, Netherlands: Elsevier 77–96.

[ref73] Lundqvist U. SvalöfWeibull A. (2005). The Swedish collection of barley mutants held at the Nordic Genebank. Barley Genet. Newslett. 35, 150–154.

[ref74] Malmberg M. M. Pembleton L. W. Baillie R. C. Drayton M. C. Sudheesh S. Kaur S.. (2018). Genotyping-by-sequencing through transcriptomics: implementation in a range of crop species with varying reproductive habits and ploidy levels. Plant Biotechnol. J. 16, 877–889. doi: 10.1111/pbi.12835 28913899 PMC5866951

[ref75] Mammadov J. Aggarwal R. Buyyarapu R. Kumpatla S. (2012). SNP markers and their impact on plant breeding. Int. J. Plant Genomics 2012, 1–11. doi: 10.1155/2012/728398 PMC3536327 23316221

[ref76] Manching H. Sengupta S. Hopper K. R. Polson S. W. Ji Y. Wisser R. J. (2017). Phased genotyping-by-sequencing enhances analysis of genetic diversity and reveals divergent copy number variants in maize. G3 7, 2161–2170. doi: 10.1534/g3.117.042036 28526729 PMC5499125

[ref77] MardisE. R. (2008). The impact of next-generation sequencing technology on genetics. Trends Genet. 24, 133–141. doi: 10.1016/j.tig.2007.12.00718262675

[ref78] Marquez-Cedillo L. A. Hayes P. M. Jones B. L. Kleinhofs A. Legge W. G. Rossnagel B. G.. (2000). QTL analysis of malting quality in barley based on the doubled-haploid progeny of two elite north American varieties representing different germplasm groups. Theor. Appl. Genet. 101, 173–184. doi: 10.1007/s001220051466

[ref79] Mascher M. Gundlach H. Himmelbach A. Beier S. Twardziok S. O. Wicker T.. (2017). A chromosome conformation capture ordered sequence of the barley genome. Nature 544, 427–433. doi: 10.1038/nature22043 28447635

[ref80] Mascher M. Muehlbauer G. J. Rokhsar D. S. Chapman J. Schmutz J. Barry K.. (2013). Anchoring and ordering NGS contig assemblies by population sequencing (POPSEQ). Plant J. 76, 718–727. doi: 10.1111/tpj.12319 23998490 PMC4298792

[ref81] Mathelier A. Shi W. Wasserman W. W. (2015). Identification of altered cis-regulatory elements in human disease. Trends Genet. 31, 67–76. doi: 10.1016/j.tig.2014.12.003 25637093

[ref82] Mayer K. F. X. Waugh R. Langridge P. Close T. J. Wise R. P. Graner A.. (2012). A physical, genetic and functional sequence assembly of the barley genome. Nature 491, 711–716. doi: 10.1038/nature11543 23075845

[ref83] Mercer T. R. Clark M. B. Crawford J. Brunck M. E. Gerhardt D. J. Taft R. J.. (2014). Targeted sequencing for gene discovery and quantification using RNA CaptureSeq. Nat. Protoc. 9, 989–1009. doi: 10.1038/nprot.2014.058 24705597

[ref85] Milne L. Bayer M. Rapazote-Flores P. Mayer C.-D. Waugh R. Simpson C. G. (2021). EORNA, a barley gene and transcript abundance database. Sci. Data 8:90. doi: 10.1038/s41597-021-00872-4 33767193 PMC7994555

[ref86] Milner S. G. Jost M. Taketa S. Mazón E. R. Himmelbach A. Oppermann M.. (2019). Genebank genomics highlights the diversity of a global barley collection. Nat. Genet. 51, 319–326. doi: 10.1038/s41588-018-0266-x 30420647

[ref87] Money D. Gardner K. Migicovsky Z. Schwaninger H. Zhong G.-Y. Myles S. (2015). LinkImpute: fast and accurate genotype imputation for nonmodel organisms. G3 5, 2383–2390. doi: 10.1534/g3.115.021667 26377960 PMC4632058

[ref88] Munyengwa N. Le Guen V. Bille H. N. Souza L. M. Clément-Demange A. Mournet P.. (2021). Optimizing imputation of marker data from genotyping-by-sequencing (GBS) for genomic selection in non-model species: rubber tree (*Hevea brasiliensis*) as a case study. Genomics 113, 655–668. doi: 10.1016/j.ygeno.2021.01.012 33508443

[ref89] Nazzicari N. Biscarini F. Cozzi P. Brummer E. C. Annicchiarico P. (2016). Marker imputation efficiency for genotyping-by-sequencing data in rice (*Oryza sativa*) and alfalfa (*Medicago sativa*). Mol. Breed. 36:69. doi: 10.1007/s11032-016-0490-y

[ref90] Niedzicka M. Fijarczyk A. Dudek K. Stuglik M. Babik W. (2016). Molecular inversion probes for targeted resequencing in non-model organisms. Sci. Rep. 6:24051. doi: 10.1038/srep24051 27046329 PMC4820773

[ref91] Nielsen R. Paul J. S. Albrechtsen A. Song Y. S. (2011). Genotype and SNP calling from next-generation sequencing data. Nat. Rev. Genet. 12, 443–451. doi: 10.1038/nrg2986 21587300 PMC3593722

[ref92] Obsa B. T. Eglinton J. Coventry S. March T. Guillaume M. Le T. P.. (2017). Quantitative trait loci for yield and grain plumpness relative to maturity in three populations of barley (*Hordeum vulgare* L.) grown in a low rain-fall environment. PLoS One 12:e0178111. doi: 10.1371/journal.pone.0178111, PMID: 28542571 PMC5441627

[ref93] Ohnoutkova L. (2019). “Mutation breeding in barley: historical overview,” in Barley. ed. Harwood W. (New York: Springer), 7–19. 10.1007/978-1-4939-8944-7_2 30460556

[ref94] O’Rawe J. Jiang T. Sun G. Wu Y. Wang W. Hu J.. (2013). Low concordance of multiple variant-calling pipelines: practical implications for exome and genome sequencing. Genome Med. 5, 28. doi: 10.1186/gm432 23537139 PMC3706896

[ref95] Ostezan A. McDonald S. C. Tran D. T. Souza R. S. E. Li Z. (2021). Target region sequencing and applications in plants. J. Crop. Sci. Biotechnol. 24, 13–26. doi: 10.1007/s12892-020-00056-3

[ref96] Ott A. Liu S. Schnable J. C. Yeh C.-T. E. Wang K.-S. Schnable P. S. (2017). tGBS^®^ genotyping-by-sequencing enables reliable genotyping of heterozygous loci. Nucleic Acids Res. 45:e178. doi: 10.1093/nar/gkx853 29036322 PMC5716196

[ref97] Pankin A. Von Korff M. (2017). Co-evolution of methods and thoughts in cereal domestication studies: a tale of barley (*Hordeum vulgare*). Curr. Opin. Plant Biol. 36, 15–21. doi: 10.1016/j.pbi.2016.12.001 28011443

[ref98] Pasam R. K. Sharma R. Malosetti M. Van Eeuwijk F. A. Haseneyer G. Kilian B.. (2012). Genome-wide association studies for agronomical traits in a world wide spring barley collection. BMC Plant Biol. 12:16. doi: 10.1186/1471-2229-12-16 22284310 PMC3349577

[ref99] Pham A.-T. Maurer A. Pillen K. Brien C. Dowling K. Berger B.. (2019). Genome-wide association of barley plant growth under drought stress using a nested association mapping population. BMC Plant Biol. 19:134. doi: 10.1186/s12870-019-1723-0 30971212 PMC6458831

[ref100] Phan N. T. Sim S.-C. (2017). Genomic tools and their implications for vegetable breeding. Hortic. Sci. Technol. 35, 149–164. doi: 10.12972/kjhst.20170018

[ref101] Pickering R. A. (1984). The influence of genotype and environment on chromosome elimination in crosses between *Hordeum vulgare* L. × *Hordeum bulbosum* L. Plant Sci. Lett. 34, 153–164. doi: 10.1016/0304-4211(84)90138-x

[ref102] Pickering R. Hill A. Michel M. Timmerman-Vaughan G. (1995). The transfer of a powdery mildew resistance gene from *Hordeum bulbosum* L to barley (*Hordeum vulgare* L.) chromosome 2 (2I). Theor. Appl. Genet. 91, 1288–1292. 24170060 10.1007/BF00220943

[ref103] Poland J. A. Brown P. J. Sorrells M. E. Jannink J.-L. (2012). Development of high-density genetic maps for barley and wheat using a novel two-enzyme genotyping-by-sequencing approach. PLoS One 7:e32253. doi: 10.1371/journal.pone.0032253 22389690 PMC3289635

[ref104] Poland J. A. Rife T. W. (2012). Genotyping-by-sequencing for plant breeding and genetics. Plant Genome 5, 92–102. doi: 10.3835/plantgenome2012.05.0005

[ref105] Pootakham W. Sonthirod C. Naktang C. Jomchai N. Sangsrakru D. Tangphatsornruang S. (2016). Effects of methylation-sensitive enzymes on the enrichment of genic SNPs and the degree of genome complexity reduction in a two-enzyme genotyping-by-sequencing (GBS) approach: a case study in oil palm (*Elaeis guineensis*). Mol. Breed. 36:154. doi: 10.1007/s11032-016-0572-x 27942246 PMC5104780

[ref106] Pourkheirandish M. Komatsuda T. (2007). The importance of barley genetics and domestication in a global perspective. Ann. Bot. 100, 999–1008. doi: 10.1093/aob/mcm139, PMID: 17761690 PMC2759206

[ref108] Raman H. Raman R. Kilian A. Detering F. Carling J. Coombes N.. (2014). Genome-wide delineation of natural variation for pod shatter resistance in *Brassica napus*. PLoS One 9:e101673. doi: 10.1371/journal.pone.0101673 25006804 PMC4090071

[ref109] Rapazote-Flores P. Bayer M. Milne L. Mayer C.-D. Fuller J. Guo W.. (2019). BaRTv1.0: an improved barley reference transcript dataset to determine accurate changes in the barley transcriptome using RNA-seq. BMC Genomics 20:968. doi: 10.1186/s12864-019-6243-7, PMID: 31829136 PMC6907147

[ref110] Rasheed A. Hao Y. Xia X. Khan A. Xu Y. Varshney R. K.. (2017). Crop breeding chips and genotyping platforms: progress, challenges, and perspectives. Mol. Plant 10, 1047–1064. doi: 10.1016/j.molp.2017.06.008 28669791

[ref111] Romero C. C. T. Vels A. Niks R. E. (2018). Identification of a large-effect QTL associated with kernel discoloration in barley. J. Cereal Sci. 84, 62–70. doi: 10.1016/j.jcs.2018.09.011

[ref112] Ruge-Wehling B. Linz A. Habekuß A. Wehling P. (2006). Mapping of Rym16 Hb, the second soil-borne virus-resistance gene introgressed from *Hordeum bulbosum*. Theor. Appl. Genet. 113, 867–873. doi: 10.1007/s00122-006-0345-8 16838136

[ref113] Sargolzaei M. Chesnais J. P. Schenkel F. S. (2014). A new approach for efficient genotype imputation using information from relatives. BMC Genomics 15:478. doi: 10.1186/1471-2164-15-478 24935670 PMC4076979

[ref114] Sato K. (2020). History and future perspectives of barley genomics. DNA Res. 27:dsaa023. doi: 10.1093/dnares/dsaa023 32979265 PMC7532727

[ref115] Sato K. Nankaku N. Takeda K. (2009). A high-density transcript linkage map of barley derived from a single population. Heredity 103, 110–117. doi: 10.1038/hdy.2009.57 19455180

[ref116] Scheben A. Batley J. Edwards D. (2017). Genotyping-by-sequencing approaches to characterize crop genomes: choosing the right tool for the right application. Plant Biotechnol. J. 15, 149–161. doi: 10.1111/pbi.12645 27696619 PMC5258866

[ref117] Scholz M. Ruge-Wehling B. Habekuß A. Schrader O. Pendinen G. Fischer K.. (2009). Ryd4 Hb: a novel resistance gene introgressed from Hordeumbulbosum into barley and conferring complete and dominant resistance to the barley yellow dwarf virus. Theor. Appl. Genet. 119, 837–849. doi: 10.1007/s00122-009-1093-3 19585100

[ref118] Schulte D. Ariyadasa R. Shi B. Fleury D. Saski C. Atkins M.. (2011). BAC library resources for map-based cloning and physical map construction in barley (*Hordeum vulgare* L.). BMC Genomics 12:247. doi: 10.1186/1471-2164-12-247 21595870 PMC3224359

[ref119] Schulte D. Close T. J. Graner A. Langridge P. Matsumoto T. Muehlbauer G.. (2009). The international barley sequencing consortium—At the threshold of efficient access to the barley genome. Plant Physiol. 149, 142–147. doi: 10.1104/pp.108.128967 19126706 PMC2613708

[ref120] Shi F. Tibbits J. Pasam R. K. Kay P. Wong D. Petkowski J.. (2017). Exome sequence genotype imputation in globally diverse hexaploid wheat accessions. Theor. Appl. Genet. 130, 1393–1404. doi: 10.1007/s00122-017-2895-3 28378053

[ref121] Singh B. D. Singh A. K. (2015). Polymerase Chain Reaction-Based Markers. New Delhi: Springer India, 47–75.

[ref123] Sonah H. Bastien M. Iquira E. Tardivel A. Légaré G. Boyle B.. (2013). An improved genotyping by sequencing (GBS) approach offering increased versatility and efficiency of SNP discovery and genotyping. PLoS One 8:e54603. doi: 10.1371/journal.pone.0054603 23372741 PMC3553054

[ref124] Srivastava R. K. Singh R. B. Pujarula V. L. Bollam S. Pusuluri M. Chellapilla T. S.. (2020). Genome-wide association studies and genomic selection in pearl millet: advances and prospects. Front. Genet. 10:1389. doi: 10.3389/fgene.2019.01389 32180790 PMC7059752

[ref125] Stein N. Prasad M. Scholz U. Thiel T. Zhang H. Wolf M.. (2007). A 1,000-loci transcript map of the barley genome: new anchoring points for integrative grass genomics. Theor. Appl. Genet. 114, 823–839. doi: 10.1007/s00122-006-0480-2, PMID: 17219208

[ref126] Szűcs P. Blake V. C. Bhat P. R. Chao S. Close T. J. Cuesta-Marcos A.. (2009). An integrated resource for barley linkage map and malting quality QTL alignment. Plant Genome 2:134. doi: 10.3835/plantgenome2008.01.0005

[ref127] Tamba C. L. Ni Y.-L. Zhang Y.-M. (2017). Iterative sure independence screening EM-Bayesian LASSO algorithm for multi-locus genome-wide association studies. PLoS Comput. Biol. 13:e1005357. doi: 10.1371/journal.pcbi.1005357 28141824 PMC5308866

[ref128] Tewhey R. Warner J. B. Nakano M. Libby B. Medkova M. David P. H.. (2009). Microdroplet-based PCR enrichment for large-scale targeted sequencing. Nat. Biotechnol. 27, 1025–1031. doi: 10.1038/nbt.1583 19881494 PMC2779736

[ref129] Torkamaneh D. Belzile F. (2015). Scanning and filling: ultra-dense SNP genotyping combining genotyping-by-sequencing, SNP Array and whole-genome resequencing data. PLoS One 10:e0131533. doi: 10.1371/journal.pone.0131533 26161900 PMC4498655

[ref130] Torkamaneh D. Boyle B. St-Cyr J. Légaré G. Pomerleau S. Belzile F. (2020). NanoGBS: a miniaturized procedure for GBS library preparation. Front. Genet. 11:67. doi: 10.3389/fgene.2020.00067 32133028 PMC7040475

[ref131] Torkamaneh D. Laroche J. Bastien M. Abed A. Belzile F. (2017). Fast-GBS: a new pipeline for the efficient and highly accurate calling of SNPs from genotyping-by-sequencing data. BMC Bioinform. 18:5. doi: 10.1186/s12859-016-1431-9 PMC5210301 28049422

[ref132] Torkamaneh D. Laroche J. Belzile F. (2016). Genome-wide SNP calling from genotyping by sequencing (GBS) data: A comparison of seven pipelines and two sequencing technologies. PLoS One 11:e0161333. doi: 10.1371/journal.pone.0161333 27547936 PMC4993469

[ref133] Ullrich S. (2010). Barley; Production, Improvement and Uses. UK: Wiley-Blackwell.

[ref134] Vafadar Shamasbi F. Jamali S. H. Sadeghzadeh B. Abdollahi Mandoulakani B. (2017). Genetic mapping of quantitative trait loci for yield-affecting traits in a barley doubled haploid population derived from clipper × Sahara 3771. Front. Plant Sci. 8:688. doi: 10.3389/fpls.2017.00688 28769936 PMC5513936

[ref136] Wang J. Araus J. L. Wan J. (2015). Breeding to optimize agriculture in a changing world. Crop J. 3, 169–173. doi: 10.1016/j.cj.2015.05.001

[ref137] Wang D. G. Fan J. B. Siao C. J. Berno A. Young P. Sapolsky R.. (1998). Large-scale identification, mapping, and genotyping of single-nucleotide polymorphisms in the human genome. Science 280, 1077–1082. doi: 10.1126/science.280.5366.1077 9582121

[ref139] Wang Q. Sun G. Ren X. Du B. Cheng Y. Wang Y.. (2019). Dissecting the genetic basis of grain size and weight in barley (*Hordeum vulgare* L.) by QTL and comparative genetic analyses. Front. Plant Sci. 10:469. doi: 10.3389/fpls.2019.00469 31105718 PMC6491919

[ref140] Wang J. Sun G. Ren X. Li C. Liu L. Wang Q.. (2016). QTL underlying some agronomic traits in barley detected by SNP markers. BMC Genet. 17:103. doi: 10.1186/s12863-016-0409-y 27388211 PMC4936321

[ref141] Wang Y. Wang K. (2021). Genome-wide identification of DNase I hypersensitive sites in plants. Curr. Protoc. 1:e148. doi: 10.1002/cpz1.148 34101388

[ref142] Wendler N. Mascher M. Nöh C. Himmelbach A. Scholz U. Ruge-Wehling B.. (2014). Unlocking the secondary gene-pool of barley with next-generation sequencing. Plant Biotechnol. J. 12, 1122–1131. doi: 10.1111/pbi.12219 25040223

[ref143] Wetterstrand K. (2021). DNA Sequencing Costs: Data from the NHGRI Genome Sequencing Program (GSP) [Online]. Available at: www.genome.gov/sequencingcostsdata (Accessed March 31, 2022).

[ref144] Wicker T. Taudien S. Houben A. Keller B. Graner A. Platzer M.. (2009). A whole-genome snapshot of 454 sequences exposes the composition of the barley genome and provides evidence for parallel evolution of genome size in wheat and barley. Plant J. 59, 712–722. doi: 10.1111/j.1365-313x.2009.03911.x 19453446

[ref145] Wickland D. P. Battu G. Hudson K. A. Diers B. W. Hudson M. E. (2017). A comparison of genotyping-by-sequencing analysis methods on low-coverage crop datasets shows advantages of a new workflow, GB-eaSy. BMC Bioinform. 18:586. doi: 10.1186/s12859-017-2000-6 PMC5745977 29281959

[ref146] Winfield M. O. Wilkinson P. A. Allen A. M. Barker G. L. A. Coghill J. A. Burridge A.. (2012). Targeted re-sequencing of the allohexaploid wheat exome. Plant Biotechnol. J. 10, 733–742. doi: 10.1111/j.1467-7652.2012.00713.x, PMID: 22703335

[ref148] Xu Y. Xu C. Xu S. (2017). Prediction and association mapping of agronomic traits in maize using multiple omic data. Heredity 119, 174–184. doi: 10.1038/hdy.2017.27 28590463 PMC5564377

[ref149] Yang Z. Chen Z. Peng Z. Yu Y. Liao M. Wei S. (2017). Development of a high-density linkage map and mapping of the three-pistil gene (Pis1) in wheat using GBS markers. BMC Genomics 18:567. doi: 10.1186/s12864-017-3960-7 28760136 PMC5537994

[ref150] Yao X. Wu K. Yao Y. Bai Y. Ye J. Chi D. (2018). Construction of a high-density genetic map: genotyping by sequencing (GBS) to map purple seed coat color (Psc) in hulless barley. Hereditas 155:37. doi: 10.1186/s41065-018-0072-6 30473656 PMC6240233

[ref151] Yu J. Buckler E. S. (2006). Genetic association mapping and genome organization of maize. Curr. Opin. Biotechnol. 17, 155–160. doi: 10.1016/j.copbio.2006.02.003 16504497

[ref152] Yu X. Kong H. Y. Meiyalaghan V. Casonato S. Chng S. Jones E. E.. (2018). Genetic mapping of a barley leaf rust resistance gene Rph26 introgressed from *Hordeum bulbosum*. Theor. Appl. Genet. 131, 2567–2580. doi: 10.1007/s00122-018-3173-8 30178277

[ref153] Yu Y. Tomkins J. P. Waugh R. Frisch D. A. Kudrna D. Kleinhofs A.. (2000). A bacterial artificial chromosome library for barley (*Hordeum vulgare* L.) and the identification of clones containing putative resistance genes. Theor. Appl. Genet. 101, 1093–1099. doi: 10.1007/s001220051584

[ref154] Zhang M. Kimatu J. N. Xu K. Liu B. (2010). DNA cytosine methylation in plant development. J. Genet. Genomics 37, 1–12. doi: 10.1016/s1673-8527(09)60020-5 20171573

[ref155] Zheng P. Allen W. B. Roesler K. Williams M. E. Zhang S. Li J.. (2008). A phenylalanine in DGAT is a key determinant of oil content and composition in maize. Nat. Genet. 40, 367–372. doi: 10.1038/ng.85 18278045

